# Edaravone ameliorates depressive and anxiety-like behaviors via Sirt1/Nrf2/HO-1/Gpx4 pathway

**DOI:** 10.1186/s12974-022-02400-6

**Published:** 2022-02-07

**Authors:** Ruozhi Dang, Mingyang Wang, Xinhui Li, Haiyang Wang, Lanxiang Liu, Qingyuan Wu, Jianting Zhao, Ping Ji, Lianmei Zhong, Julio Licinio, Peng Xie

**Affiliations:** 1 NHC Key Laboratory of Diagnosis and Treatment on Brain Functional Diseases, The First Affiliated Hospital of Chongqing Medical University, 1 Youyi Road, Yuzhong District, Chongqing, 400016 China; 2 Department of Neurology, The First Affiliated Hospital of Chongqing Medical University, Chongqing, 400016 China; 3 College of Stomatology and Affiliated Stomatological Hospital of Chongqing Medical University, Chongqing, 401147 China; 4 Department of Neurology, Yongchuan Hospital of Chongqing Medical University, Chongqing, 402160 China; 5 Department of Neurology, Chongqing University Three Gorges Hospital, Chongqing, 404100 China; 6 Department of Neurology, Xinxiang Central Hospital, Xinxiang, 453000 China; 7 Chongqing Key Laboratory of Oral Diseases and Biomedical Sciences, College of Stomatology and Affiliated Stomatological Hospital of Chongqing Medical University, Chongqing, 401147 China; 8 Key Laboratory of Psychoseomadsy, Stomatological Hospital of Chongqing Medical University, Chongqing, 401147 China; 9 Department of Neurology, First Affiliated Hospital of Kunming Medical University, Kunming, 650032 China; 10 Department of Psychiatry and Behavioral Sciences, College of Medicine, SUNY Upstate Medical University, Syracuse, NY USA

**Keywords:** Edaravone, Depression, Anxiety, Oxidative stress, Gpx4, Ferroptosis

## Abstract

**Background:**

The inflammation and oxidative stress (OS) have been considered crucial components of the pathogenesis of depression. Edaravone (EDA), a free radical scavenger, processes strong biological activities including antioxidant, anti-inflammatory and neuroprotective properties. However, its role and potential molecular mechanisms in depression remain unclear. The present study aimed to investigate the antidepressant activity of EDA and its underlying mechanisms.

**Methods:**

A chronic social defeat stress (CSDS) depression model was performed to explore whether EDA could produce antidepressant effects. Behaviors tests were carried out to examine depressive, anxiety-like and cognitive behaviors including social interaction (SI) test, sucrose preference test (SPT), open field test (OFT), elevated plus maze (EPM), novel object recognition (NOR), tail suspension test (TST) and forced swim test (FST). Hippocampal and medial prefrontal cortex (mPFC) tissues were collected for Nissl staining, immunofluorescence, targeted energy metabolomics analysis, enzyme-linked immunosorbent assay (ELISA), measurement of MDA, SOD, GSH, GSH-PX, T-AOC and transmission electron microscopy (TEM). Western blotting (WB) and quantitative real-time polymerase chain reaction (qRT-PCR) detected the Sirt1/Nrf2/HO-1/Gpx4 signaling pathway. EX527, a Sirt1 inhibitor and ML385, a Nrf2 inhibitor were injected intraperitoneally 30 min before EDA injection daily. Knockdown experiments were performed to determine the effects of Gpx4 on CSDS mice with EDA treatment by an adeno-associated virus (AAV) vector containing miRNAi (Gpx4)–EGFP infusion.

**Results:**

The administrated of EDA dramatically ameliorated CSDS-induced depressive and anxiety-like behaviors. In addition, EDA notably attenuated neuronal loss, microglial activation, astrocyte dysfunction, oxidative stress damage, energy metabolism and pro-inflammatory cytokines activation in the hippocampus (Hip) and mPFC of CSDS-induced mice. Further examination indicated that the application of EDA after the CSDS model significantly increased the protein expressions of Sirt1, Nrf2, HO-1 and Gpx4 in the Hip. EX527 abolished the antidepressant effect of EDA as well as the protein levels of Nrf2, HO-1 and Gpx4. Similarly, ML385 reversed the antidepressant and anxiolytic effects of EDA via decreased expressions of HO-1 and Gpx4. In addition, Gpx4 knockdown in CSDS mice abolished EDA-generated efficacy on depressive and anxiety-like behaviors.

**Conclusion:**

These findings suggest that EDA possesses potent antidepressant and anxiolytic properties through Sirt1/Nrf2/HO-1/Gpx4 axis and Gpx4-mediated ferroptosis may play a key role in this effect.

**Supplementary Information:**

The online version contains supplementary material available at 10.1186/s12974-022-02400-6.

## Background

Major depression disorder (MDD), a common, debilitating psychiatric disorder, affects approximately 17% of the population at some point in life [1, 2]. Although there are available antidepressants, most of the pharmacologic interventions take weeks to months to produce effects and have low response rates [3, 4]. Furthermore, some striking studies have found that ketamine can produce rapid antidepressant effects, and its s-isomer, esketamine has been approved by the Food and Drug Administration (FDA) to treat adults with treatment-resistant depression [5, 6]. Although groundbreaking, some serious side effects, dissociative, psychotomimetic action and abuse potential limit its wide application. Therefore, novel therapies with fewer adverse effects and higher efficacy are urgently needed.

Accumulating evidence demonstrated a close link between oxidative stress (OS) and MDD [7, 8]. In general, OS is defined as an imbalance between the oxidation and antioxidant capacity, involving reactive oxygen species (ROS) and reactive nitrogen species (RNS) [9]. ROS is involved in glutamate-dependent long-term potentiation (LTP) and moderate ROS is necessary for the growth and development of neurons [8]. Abundant studies showed that MDD was accompanied by decreased antioxidant and increased ROS status [8, 10, 11]. Moreover, preclinical and clinical evidence on OS and antioxidant effects of antidepressants addressed that they can scavenge ROS and RNS by scavenging free radicals and inhibiting OS pathways [11]. This process will protect neurons from the effect of OS and alleviate depression. In addition, preclinical and clinical studies have also shown that inflammation plays a crucial role in depression [12–14]. Increased pro-inflammatory cytokines, including IL-1β, IL-6 and TNF-α were found in MDD patients, postmortem brains with MDD and rodent studies [15–17]. Silent information regulator 2 homolog 1 (Sirt1), a NAD^+^ dependent protein deacetylase, regulates acetylation of specific transcription factors, proteins and is involved in abundant functions including energy metabolism, stress responses, inflammation and redox homeostasis [18, 19]. Specifically, recent studies have shown that Sirt1 plays a crucial role in depression and regulates downstream transcription factor including nuclear factor erythroid 2-related factor 2 (Nrf2) and NF-κB to prevent OS and inflammation damage [20–22]. Nrf2 is a primary transcription factor in the regulation of antioxidant response and has emerged as a potential therapeutic target for inflammatory disorders [23]. Dysregulation of Nrf2 leads to the decrease of antioxidants and detoxifying enzymes, which is involved in the pathogenesis of depression [24]. Heme oxygenase-1 (HO-1), a critical downstream target stress inducible protein of Nrf2, also exerts antioxidant stress and anti-inflammatory effects [25].

Ferroptosis, a recently discovered non-caspase-dependent form of programmed cell death, is characterized by the production of iron-dependent lipid ROS and associated with OS and inflammation [26, 27]. It differs from other types of cell death, such as apoptosis, autophagy, necrosis, and pyroptosis in morphological, physiological and genetically characteristics [28]. Both Nrf2 and HO-1 could be inducible and participate in the synthesis of glutathione peroxidase 4 (Gpx4), which is the first discovered central inhibitor of ferroptosis [29]. Gpx4, a lipid repair enzyme, plays a key role in the regulation of ferroptosis. Inhibition of Xc^−^ leads to depletion of cysteine and impaired the function of Gpx4, which eventually leads to ferroptosis [30]. Other studies have shown that Gpx4 can be up-regulated by pro-inflammatory cytokines and overexpression of Gpx4 can prevent cell death from oxidative damage [31–33]. To the best of our knowledge, there is still no systematic research on the relation between ferroptosis and depression.

Edaravone (3-methyl-1-phenyl-2-pyrazolin-5-one, EDA), a free radical scavenger, processes strong biological activities including antioxidant, neuroprotective and anti-inflammatory effects [34]. It is commonly used to treat acute ischemic stroke and acute cerebral infarction. Moreover, EDA is effective in altering amyotrophic lateral sclerosis (ALS) progression, which is the second approved drug for treatment of ALS [35]. Recent studies have found that EDA exerts antioxidant and anti-inflammatory effects by Nrf2/HO-1 signaling pathway in asthma and cerebral infarction [36]. Interestingly, only a few studies have addressed EDA can attenuate depressive-like behavior [37, 38]. However, the detailed molecular mechanisms remain unclear. Accordingly, we hypothesized that EDA might ameliorate depressive and anxiety-like behaviors via Sirt1/Nrf2/HO-1/Gpx4 pathway.

To investigate this hypothesis, we established chronic stress defeated stress (CSDS) depression mice model and found EDA ameliorated depressive, anxiety-like behaviors and neuronal loss, affected energy metabolism, inhibited microglial activation, OS damage and pro-inflammatory cytokines (IL-1β, IL-6 and TNF-α) activation, attenuated astrocyte dysfunction and suppressed ferroptosis and inflammation response by regulating the Sirt1/Nrf2/HO-1/Gpx4 pathway. Furthermore, the pivotal role of Gpx4-mediated ferroptosis in EDA-related antidepressant and anxiolytic effects was confirmed using Gpx4 knockdown virus.

## Materials and methods

### Animals and drugs treatment

Male C57BL/6J mice (aged 7–8 weeks) and retired male CD-1 mice (aged 16–20 weeks) were obtained from the Experimental Animal Centre of Chongqing Medical University (Chongqing, China). The experimental animals were housed in cages under a 12 h light/12 h dark cycle (lights on at 8:00 a.m.), 60 ± 5% humidity, and a temperature of 23 ± 1 °C with access to water and food freely. All experimental procedures were conducted in accordance with the Ethics Committee of Chongqing Medical University. EDA was purchased from Sigma-Aldrich (St. Louis, USA) and was dissolved in Vehicle (NaCl, 0.9%) at a dosage of 10 mg/kg. EX527 (a Sirt1 inhibitor) and ML385 (a Nrf2 inhibitor) were obtained from MedChemExpress (New Jersey, USA). The doses of these three drugs were determined according to previous studies [37–40].

The study was conducted into four experiments.

**Fig. 1:**
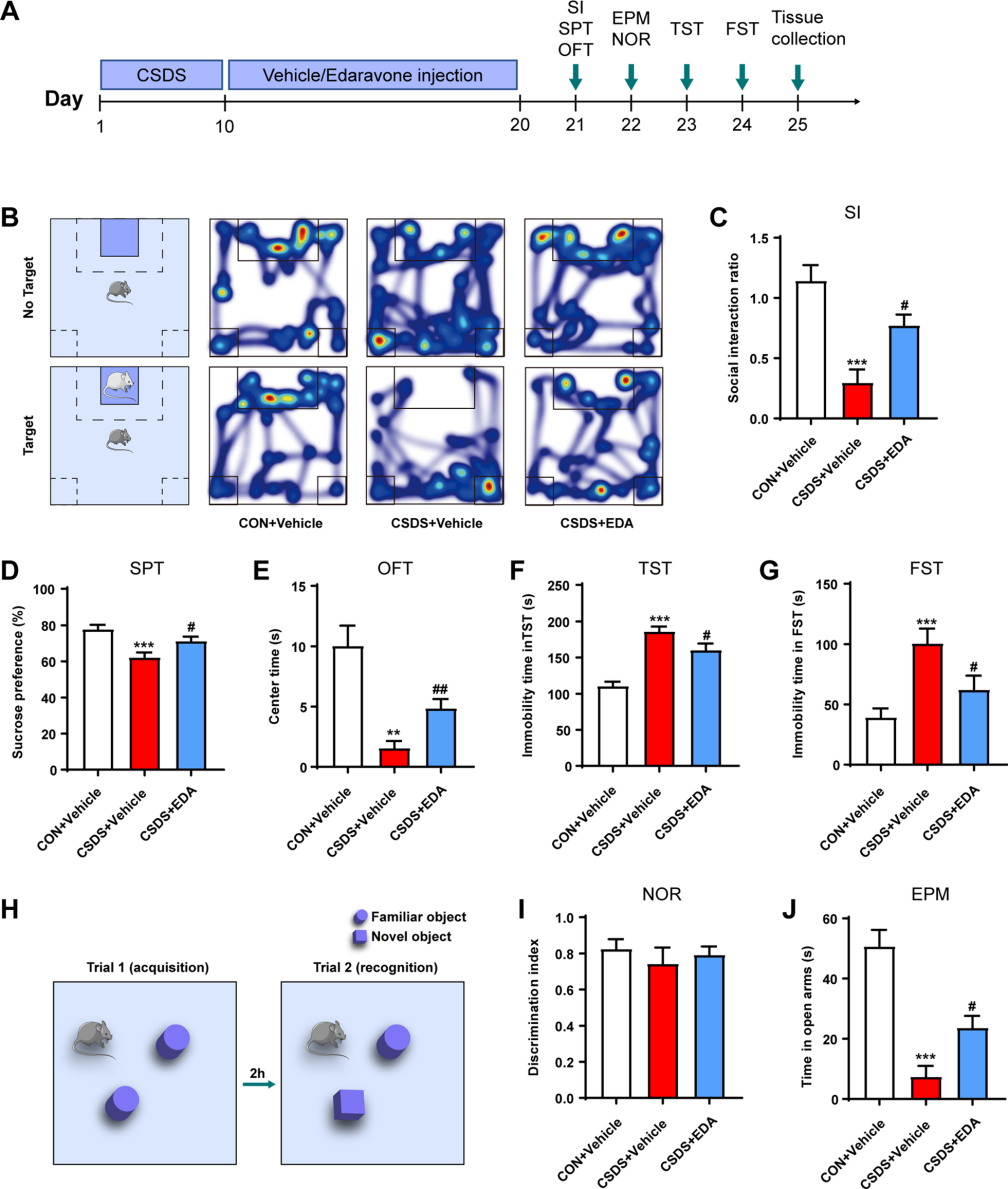
Effects of EDA on CSDS-induced depressive and anxiety-like behaviors. **A** Schematic representation of the CSDS procedure and treatments in mice. **B** Heat maps of path tracing in the social interaction test after EDA administration. **C** Social interaction test. **D** Sucrose preference test. **E** Open field test. **F** Tail suspension test. **G** Forced swimming test. **H**, **I** Novel object recognition test. **J** Elevated plus maze test. All the data are expressed as mean ± SEM (*n* = 10 per group). **p < 0.01, ***p < 0.001 versus the CON + Vehicle group. ^#^*p* < 0.05, ^##^*p* < 0.01 versus the CSDS + Vehicle group

In the first experiment, mice were randomly divided into three groups (10 each group): Control (CON) + Vehicle, CSDS + Vehicle and CSDS + EDA [10 mg/kg, intraperitoneally (i.p.)]. The experiment schedule is shown in Fig. 1A. After 10 day CSDS model, mice were administered intraperitoneally (i.p.) daily injections of EDA or Vehicle for 10 days followed by behavioral tests and tissue collection.

In the second experiment, mice were randomly divided into the following groups (*n* = 8 per group): CON, CSDS, CSDS + EDA, CSDS + EDA + EX527 [10 mg/kg/d, intraperitoneally (i.p.)]. The experiment schedule is shown in Fig. 12A. EX527 was administered in CSDS-induced mice 30 min before the administration of EDA.

In the third experiment, mice were randomly divided into the following groups (*n* = 8 per group): CON, CSDS, CSDS + EDA, CSDS + EDA + ML385 [30 mg/kg/d, intraperitoneally (i.p.)]. The experiment schedule is shown in Fig. 14A. ML385 was administered in CSDS exposed mice 30 min before the administration of EDA.

To explore the Gpx4 role in CSDS-induced depression and anxiety, animals were randomized divided into six groups (10 each group): CON, CSDS, CSDS + Vehicle, CSDS + EDA, CSDS + EDA + CON-miRNAi and CSDS + EDA + Gpx4-miRNAi. The experiment schedule is shown in Fig. 16A.

### Chronic social defeat stress (CSDS)

The CSDS paradigm was conducted as described previously [41]. Retired male CD-1 mice were screened for 3 consecutive days and the aggressors were selected according to the following criteria: (i) CD1 mice attacked the C57BL/6J mice for 2 consecutive days and (ii) the latency to the initial attack on C57BL/6J mice was under 60 s (s). For 10 consecutive days, mice were placed in the home cage of CD1 and were defeated for 5 min. After physical defeat, a perforated divider separated them for 24 h until the next physical defeat. Each day, intruder mice always faced a different resident. After 10 days of CSDS, retired male CD-1 mice and experimental mice were singly housed and performed 24 h later for the social interaction (SI) test.

SI test was composed of two stages. In the first stage, the experimental mice were allowed to explore an open field arena (44 cm × 44 cm × 30 cm) with an empty plastic box (10 cm × 7 cm × 18 cm) for 2.5 min. In the second stage, a novel, aggressive CD-1 mouse was placed in the plastic box and was recorded for 2.5 min. The time of mice spent in the interaction zone (IZ) surrounding the plastic box was recorded and analyzed (Ethovision, Noldus, The Netherlands). For SI test, an SI ratio was calculated. SI ratio = time spent in interaction zone with a CD1 mouse/time spent in interaction zone without a CD1 mouse. Susceptible mice were defined by SI < 1, whereas resilient mice were defined by SI > 1. All mice used in the first experiment were susceptible mice.

### Sucrose preference test (SPT)

Before the experiment, mice were habituated with two identical water bottles for 3 days. Each mouse was water and food-deprived for 24 h and then provided with 1% water and sucrose solution. After 12 h, the bottles were weighted and the sucrose preference was calculated as [sucrose water intake/(sucrose water intake + pure water intake)] × 100%.

### Open field test (OFT)

A plain, 44 cm × 44 cm × 30 cm open field arena was used to assess the locomotor activity and anxiety-like behavior [42]. After a 30 s habituation, the total distance traveled, time spent in center arena (14.7 cm × 14.7 cm) was recorded in a 5 min session.

### Elevated plus maze (EPM)

The EPM test was used to measure anxiety-related behavior in rodents [43]. The apparatus was composed of two closed arms (30 cm × 6 cm × 15 cm) and two open arms (30 cm × 6 cm). Animals were placed in the EPM to explore for 5 min and time spent in closed and open arms was calculated.

### Tail suspension test (TST)

Mice were suspended by the tails by using adhesive tape 1 cm from the tip of the tail and 15 cm above the ground. Small plastic tubes were attached to tails to make sure mice could not climb. The tests lasted for 6 min and the immobility time was measured and analyzed during the final 4 min.

### Forced swim test (FST)

Mice were placed individually into a transparent glass cylinder (diameter 15 cm, height 30 cm) filled with 15 cm depth of water (23 ± 1 °C). The animals were placed in the cylinders for 6 min and the immobility time was calculated during the last 4 min.

### Novel object recognition (NOR)

The NOR test was performed according to previous protocols [44]. Briefly, the test was performed in an open field arena (44 cm × 44 cm × 30 cm). Objects were fixed to the open field arena and had different shapes, sizes and textures. The NOR test consists of two stages. During a 5 min acquisition phase, the animals were placed at the center of the arena in the presence of two identical objects (height: 12 cm; base diameter: 7 cm). After 2 h, a 5 min retrieval phage was conducted and one of the two familiar objects was replaced by a novel object (6.5 cm × 6.5 cm × 6.5 cm). The time spent exploring familiar and novel objects was recorded and analyzed. The apparatus was cleaned with 75% ethanol after each test. The “discrimination index” was calculated by the following [(novel object time)/(novel object time + familiar object time)]. Object exploration was defined when the mice touch the object with its nose within 2 cm or less, and actively explore the objects.

### Nissl staining

10-μm coronal cryosections were stained with Nissl staining solution (Beyotime, C0117, Shanghai, China) for 5 min at 37 °C. Then samples were washed using 95% ethyl alcohol for 5 min and dried. Sections were then washed twice in xylene for 5 min. After being sealed with neutral balsam, the slides were observed under an optical microscope (Axio Image A2; Carl Zeiss, Germany) by a blinded investigator.

### Immunofluorescence and image analysis

Immunofluorescence staining was performed on frozen coronal sections of mice brains. After post-fixation and concentration dehydration, the brains were cut into 20 μm thick sections. Then the slices were washed in PBS for 5 min three times, blocked with 5% bovine serum albumin (BSA) and 0.2% Triton X-100 for 2 h at room temperature. After blocking, the sections were incubated with primary antibodies overnight at 4 °C, and then sequentially incubated with the secondary antibodies for 2 h at room temperature. Primary antibodies include: Rabbit anti-Iba-1 (1:500, Wako, 019-19741), Goat anti-GFAP (1:500, Abcam, ab53554), Rabbit anti-TREM2 (1:150, Proteintech, 13483-1-AP), Rabbit anti-Connexin 30 (1:200, Abcam, ab200866), Rabbit anti-Connexin 43 (1:100, Thermo, 710700). The nuclei were counterstained with DAPI for 10 min. Fluorescent images were captured with a fluorescence microscope (VS200; Olympus, Japan) and a confocal microscope (A1R, Nikon, Japan). The number of cells was counted manually containing the intact hippocampus (Hip) and medial prefrontal cortex (mPFC) from 3 mice in each group. Branch length and branch number of Iba-1 and GFAP positive cells were measured using Image-Pro Plus 6.0 (Media Cybernetics, USA) and five micrographs for each mouse were analyzed. Areas of CX43 and CX30 staining were analyzed using Image J software.

### Transmission electron microscopy (TEM)

Mice were anesthetized and perfused with phosphate-buffered solution (PBS) followed by 4% paraformaldehyde. Small Hip and mPFC tissues (1 mm^3^) were quickly dissected and post-fixed overnight at 4 °C using 2.5% glutaraldehyde. Tissues were embedded and cut into along the coronal plane at a thickness of 60–80 nm. The mitochondrial morphology was detected by transmission electron microscope (JEM-1400 PLUS, Japan) by a blinded pathologists.

### Measurement of MDA, SOD, GSH, GSH-PX and T-AOC

The activities of the antioxidant enzymes of malondialdehyde (MDA), the superoxide dismutase (SOD), reduced glutathione (GSH), the glutathione peroxidase (GSH-PX) and total antioxidant capacity (T-AOC) within Hip and mPFC tissues were measured with MDA activity assay kit (No. A003-1-2), SOD activity assay kit (No. A001-3-2) and GSH activity assay kit (No. A006-2-1), GSH-PX activity assay kit (No. A005-1-2) and T-AOC assay kit (S0121, Beyotime, China). MDA, SOD, GSH and GSH-PX kits were purchased from Jiancheng Inc. (Nanjing, China). In addition, it was necessary to pre-treat the brain tissues before measuring the above assay kits. Briefly, the tissues were weighted and PBS (pH 7.2–7.4, 0.01 mol/L) was added at a 1:9 proportion (MDA, SOD, GSH and GSH-PX kits) and a 1:5 proportion for T-AOC. Next, the samples were treated with tissue lyser (Jingxin, China), followed  by centrifuging the homogenate at 12,000 rpm for 10 min at 4 °C, and collecting the supernatant to measure.

### LC–MS/MS analysis

LC–MS/MS was conducted according to previous studies [45]. Briefly, 30 mg brain tissues were preconditioned with 200 μl cool ultrapure water for homogenization. Then 800 μl methanol/acetonitrile (1:1, v/v) were added and the mixture was sonicated twice for 30 min and placed 1 h at − 20 °C to precipitate proteins. After centrifugation, the supernatants were dried and collected. The analysis of supernatants was conducted with Agilent 1290 Infinity LC chromatography system and AB SCIEX 5500 QTRAP mass spectrometer.

### Western blotting (WB)

The Hip and mPFC tissues were dissociated by radio-immunoprecipitation assay solution containing protease inhibitors and phosphatase inhibitors. The proteins in the samples were run on 10% SDS polyacrylamide gel and then transferred to polyvinylidene fluoride membrane (Millipore, USA). The proteins were incubated with Rabbit anti-Sirt1 (1:1000, Abcam, ab189494), Rabbit anti-Nrf2 (1:1000, Proteintech, 16396-1-AP), Rabbit anti-HO-1 (1:1000, Proteintech, 10701-1-AP), Rabbit anti-Gpx4 (1:3000, Abcam, ab125066), Rabbit anti-TLR4 (1:1000, Abcam, ab217274), Rabbit anti-NF-κB p65 (phospho S536) (1:1000, Abcam, ab76302), Rabbit anti-NF-κB p65 (1:1000, Cell Signaling, 8242) and Mouse anti-GAPDH (1:10,000, Abcam, ab8245) overnight at 4 °C, and secondary anti-mouse HRP-conjugated (1:10,000, Bio-rad, 170-6516) or secondary anti-rabbit HRP-conjugated antibodies (1:10,000, Bio-rad, 170-6515) were incubated for 2 h at room temperature. The signals were visualized using ECL chemiluminescence reagent (Beyotime; US Everbright Inc.).

### Quantitative real-time polymerase chain reaction (qRT-PCR)

Total RNA in Hip and mPFC lysates of mice was extracted with TRIzol reagent (Invitrogen, Carlsbad, USA) and reverse transcribed into cDNA by PrimeScript RT reagent Kit (Takara, Japan) following the manufacturer’s guides. Relative mRNA expression levels were performed with SYBR Green detection system (Roche, Germany). All samples were performed in triplicate and normalized with β-actin mRNA level. The primer sequences are listed in Table 1.

**Table 1:** Primer sequences used in the qRT-PCR

Gene	Primer sequences
Sirt1	Forward: 5′-GCTGACGACTTCGACGACG-3′ Reverse: 5′-TCGGTCAACAGGAGGTTGTCT-3′
Nrf2	Forward: 5′-TCTTGGAGTAAGTCGAGAAGTGT-3′ Reverse: 5′-GTTGAAACTGAGCGAAAAAGGC-3′
HO-1	Forward: 5′-AAGCCGAGAATGCTGAGTTCA-3′ Reverse: 5′-GCCGTGTAGATATGGTACAAGGA-3′
Gpx4	Forward: 5′-CTGCTCTTCCAGAGGTCCTG-3′ Reverse: 5′-GAGGTGTCCACCAGAGAAGC-3′
β-Actin	Forward: 5′-ACTGCCGCATCCTCTTCCT-3′ Reverse: 5′-TCAACGTCACACTTCATGATGGA-3′

### Enzyme-linked immunosorbent assay (ELISA)

The levels of TREM2, GFAP, IL-1β, IL-6 and TNF-α in the Hip and mPFC were measured by ELISA kits (Jianglai Biological, Shanghai, China). The tissues were  weighed, and PBS was added at a 1:9 proportion and homogenized. Then, the homogenate was centrifuged at 5000 rpm for 15 min, and collecting the supernatant to measure. According to the instructions, 100 μl standard/sample was added and incubated for 1 h at 37 °C. Then, biotin antibody was added into each well and incubated for 1 h at 37 °C. The plate was then washed with washing buffer and streptavidin–HRP was added for 30 min at 37 °C. Next, TMB substrate was added to each well and kept away from light for 15 min at 37 °C after washing. Finally, stop solution was added and the optical density was measured at 450 nm.

### Stereotaxic virus injection

Mice were anesthetized with isoflurane and then placed on a stereotaxic apparatus (RWD, Shenzhen, China). Virus was injected using Nanoject II (Drummond Scientific, PA) via a micro pipette. 3 min after pipette insertion, small amounts of virus were multiple injected at 30 s intervals (13 nl/s). After waiting for 10 min, the pipette was slowly retracted. 2 μl AAV2/8-CMV-EGFP-miRNAi(Gpx4) or AAV2/8-CMV-EGFP-miRNAi(NC) (Taitool, Shanghai, China) was bilaterally injected into the Hip (coordinates, bregma: AP = − 1.70 mm; ML =  ± 1.25 mm; DV = − 1.20 mm, bregma: AP = − 3.15 mm; ML =  ± 2.94 mm; DV = − 3.62, − 2.25 mm). Behavioral tests were commenced 4 weeks after viral injection.

### Statistical analysis

Data were presented as mean ± SEM and analyzed with GraphPad Prism 8.0 or the statistical program SPSS 17.0. The normality of data was assessed individually and non-parametric test were used when *p* < 0.05. Homogeneity of variance was be verified by Brown–Forsythe test, when *p* < 0.05, data were analyzed by one way analysis of variance (ANOVA) followed by Dunnett’s T3 post hoc test. Statistical comparisons among groups were performed by using ANOVA followed by Tukey’s test or Fisher’s LSD (data for energy metabolites in Fig. 9) post hoc test for normally distributed data and Kruskal–Wallis test with multiple comparisons for non-normally distributed data. *p* < 0.05 was regarded as significant.

## Results

### EDA ameliorates depression and anxiety-related behaviors after CSDS exposure

To investigate the effect of EDA on depression, we exposed mice to a well-established animal model of depression, CSDS, and subsequently performed multiple behavioral tests including SI, SPT, OFT, NOR, EPM, TST, and FST (Fig. 1A). As shown in Fig. 1B, C, mice exposed to CSDS showed significantly decreased SI ratio. However, intraperitoneal injection of EDA improved the SI ratio as compared to the CSDS + Vehicle group. In SPT, the consumption of sucrose was significantly reduced in CSDS exposed mice compared with the control mice. Nevertheless, EDA intervention greatly reversed CSDS-induced reduction in sucrose preference, which reflected anhedonia (Fig. 1D). Next, mice, exposed to an inescapable aversive environment TST and FST, initially violent escape but then transition to a passive coping (PC) state [46]. This state can be intervened by pharmacological approach and may be associated with the pathological motivational impairments in MDD [47]. We found that CSDS + Vehicle animals exhibited robust increase immobility in TST and FST. As expected, CSDS + EDA mice exhibited significant decreases in time spent immobile compared to CSDS + Vehicle mice (Fig. 1F, G). These results consistently indicated that EDA elicits antidepressant effect in mice.

In addition, we employed OFT and EPM to evaluate anxiety-like behavior. The CSDS + Vehicle mice exhibited remarkable anxiety-like behavior relative to the controls, as indicated by less time spent in the center zone of OFT and the open arms in the EPM. Nevertheless, this reduction was markedly reversed by the administration of EDA, indicating EDA was effective in ameliorating CSDS-evoked anxiety (Fig. 1E, J).

Previous study has observed oral administration of EDA ameliorated the Alzheimer's disease (AD)-like pathologies and memory deficits of the mice [48]. Accordingly, we carried out the NOR test to determine whether EDA affects the recognition memory of mice (Fig. 1H, I). Interestingly, there was no difference in time spent with the novel object among all groups, displayed no impairment of object memory retention.

### EDA treatment significantly alleviates neuronal injury

**Fig. 2:**
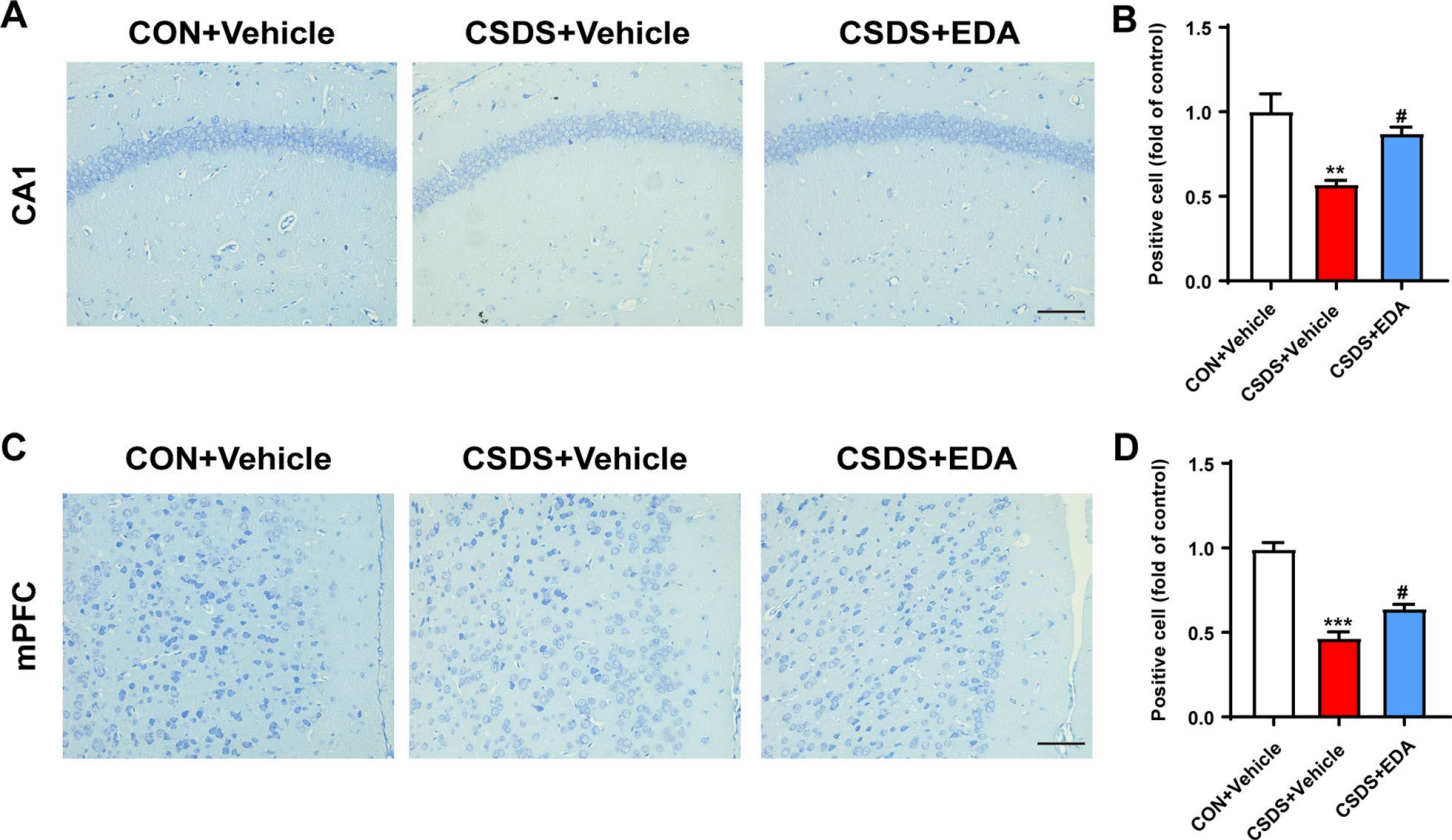
Influence of EDA on neuronal death in the Hip and mPFC. **A**, **B** Nissl staining in the hippocampal CA1 subregion and its statistical analysis. **C**, **D** Nissl staining in the mPFC and its statistical analysis. Data are presented as mean ± SEM (*n* = 3 per group). Scale bar, 50 μm. **p < 0.01, ***p < 0.001 versus the CON + Vehicle group. ^#^*p* < 0.05 versus the CSDS + Vehicle group

MDD was thought to be primarily triggered through neuronal dysfunction in the Hip and mPFC [49]. Based on existing evidence, Nissl staining was employed to observe neuronal morphological changes in the Hip and mPFC. As shown in Fig. 2A, C, neurons in the hippocampal CA1 and mPFC regions were arrayed regularly and compactly and Nissl bodies were clear in the CON + Vehicle group. Whereas, CSDS-evoked mice have increased damaged neurons, which were irregular and sparsely distributed as well as Nissl body disintegrated compared with the CON + Vehicle group. The number of Nissl-positive cells was observably reduced compared with the CON + Vehicle group. After EDA administration, the decrease of Nissl-positive neurons and neuronal damage were ameliorated (Fig. 2B, D). These data indicated that EDA could attenuate CSDS-induced neuronal damage.

### Administration of EDA inhibits microglial activation and attenuates astrocyte dysfunction in the CSDS exposed mice

**Fig. 3:**
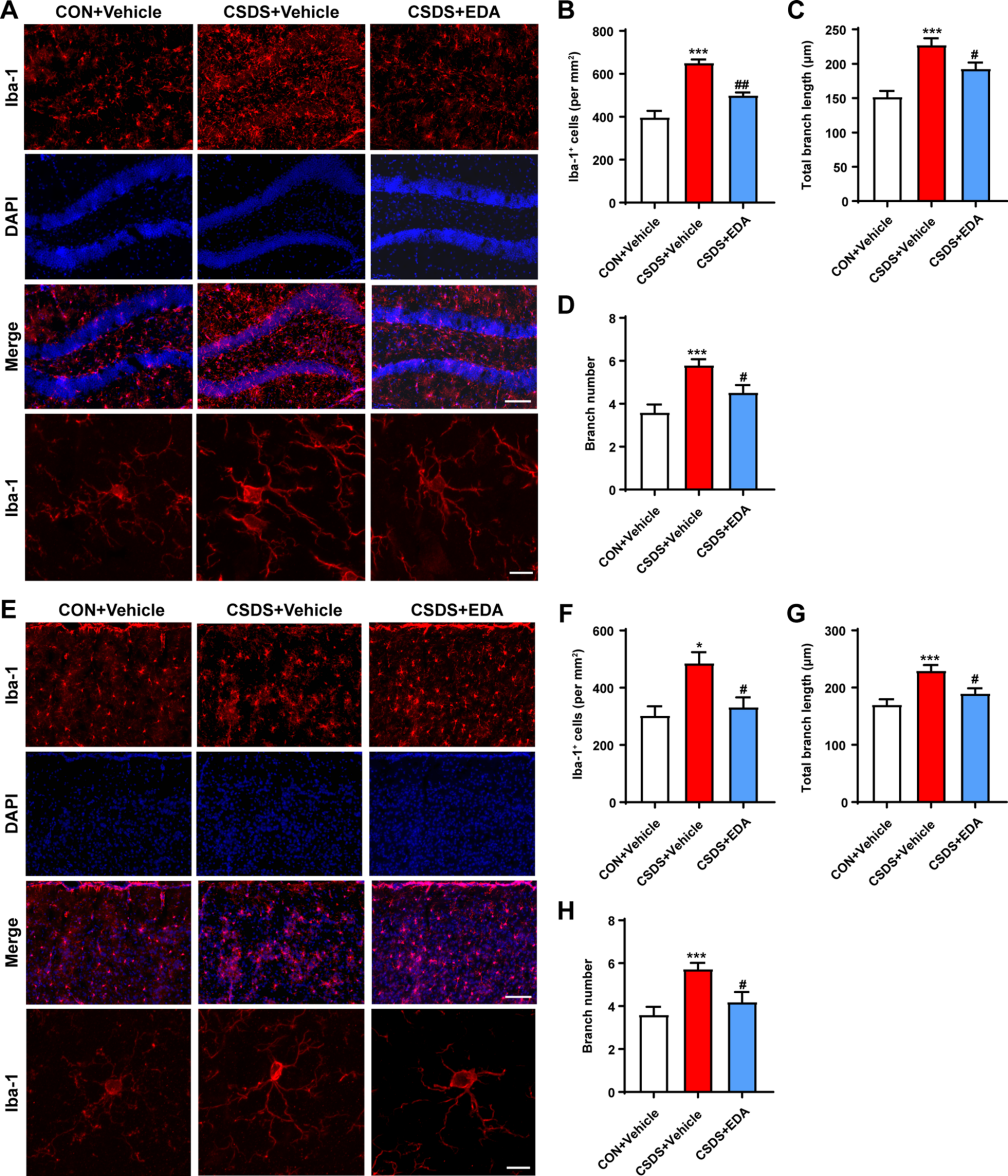
Effects of EDA on microglial activation in the CSDS mice Hip and mPFC. **A** EDA inhibited microglial activation in the CSDS mice Hip. Representative images of microglia immunostaining for Iba-1. Scale bars, 100 μm (upper panel) and 10 μm (lower panel). **B** Quantification of Iba-1 positive cells per mm^2^ in the mice Hip. **C** Total branch length. **D** Branch number. **E** EDA inhibited microglial activation in the CSDS mice mPFC. Representative images of microglia immunostaining for Iba-1. Scale bars, 100 μm (upper panel) and 10 μm (lower panel). **F** Quantification of Iba-1 positive cells per mm^2^ in the mPFC. **G** Total branch length. **H** Branch number. All the data are presented as mean ± SEM (*n* = 3 mice/group, 15 cells/group). *p < 0.05, ***p < 0.001 versus the CON + Vehicle group. ^#^*p* < 0.05, ^##^*p* < 0.01 versus the CSDS + Vehicle group

**Fig. 4:**
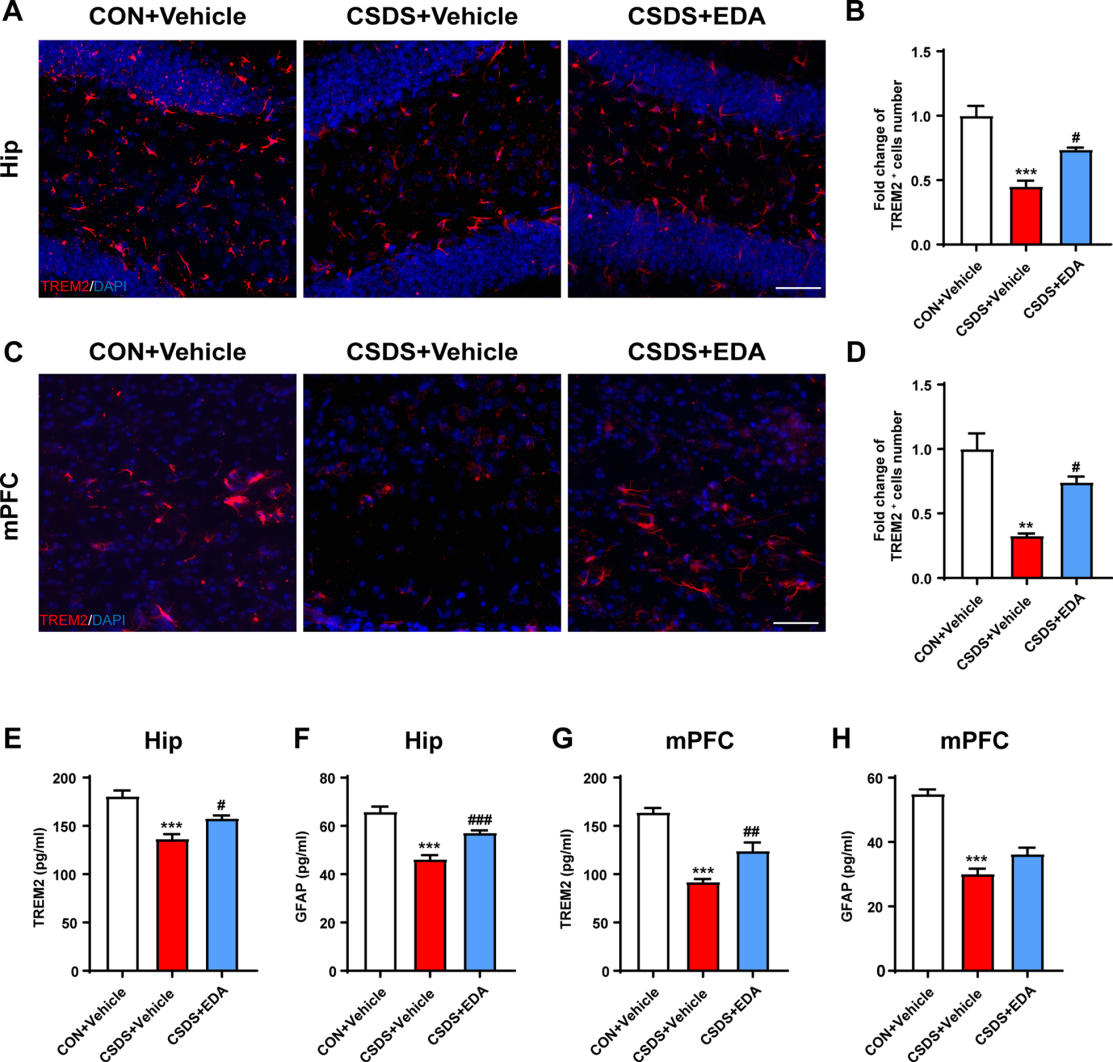
Administration of EDA increased the expression of TREM2 and GFAP. **A**, **B** Immunofluorescence staining of TREM2 in the Hip. Scale bar, 50 μm. **C**, **D** Immunofluorescence staining of TREM2 in the mPFC. Scale bar, 50 μm. **E**, **F** Concentrations of Trem2 and GFAP in the Hip. **G**, **H** Concentrations of TREM2 and GFAP in the mPFC. Data are presented as mean ± SEM (*n* = 3 per group for immunostaining experiments, *n* = 6 mice for ELISA). **p < 0.01, ***p < 0.001 versus the CON + Vehicle group. ^#^*p* < 0.05, ^##^*p* < 0.01, ^###^*p* < 0.001 versus the CSDS + Vehicle group

Neuroinflammation is tightly associated with the onset of depression [50, 51]. Given that microglia and astrocytes play an essential role in neuroinflammation and undergo abnormally structural and functional changes in the Hip and mPFC in response to chronic stress, we first examined the effect of EDA on the distribution and morphology of microglia in the Hip and mPFC using ionized calcium-binding adaptor molecule 1 (Iba-1) staining (Fig. 3A, E). The hippocampal and mPFC microglia exhibited striking activation morphology after CSDS exposure, as indicated by increased cell number, total branch length and branch number. These effects were significantly alleviated by EDA administration (Fig. 3B–D, F–H). The triggering receptor expressed on myeloid cells 2 (TREM2) is an innate immune receptor and uniquely expressed by microglia in the brain [52]. Mounting evidence have indicated TREM2 regulates inflammatory responses mediated by microglia [53, 54]. To convincingly verify the key role of microglial activation in CSDS-induced mice, the TREM2 was detected by Immunofluorescence and ELISA. As shown in Fig. 4A, B, we found that the expression of TREM2 was dramatically reduced in the CSDS + Vehicle mice compared with that of CON + Vehicle mice in the Hip. After treatment with EDA, the TREM2 expression level was markedly up-regulated in the CSDS mice. Similar phenotype was also identified in the mPFC region (Fig. 4C, D). In addition, decreased protein level of TREM2 induced by CSDS was remarkably increased by EDA treatment in the Hip and mPFC through ELISA kit (Fig. 4E, G).

Since CSDS mice experience neuroinflammation leading to astrocyte dysfunction, we then examined the effect of EDA on astrocyte morphology and functional change.

**Fig. 5:**
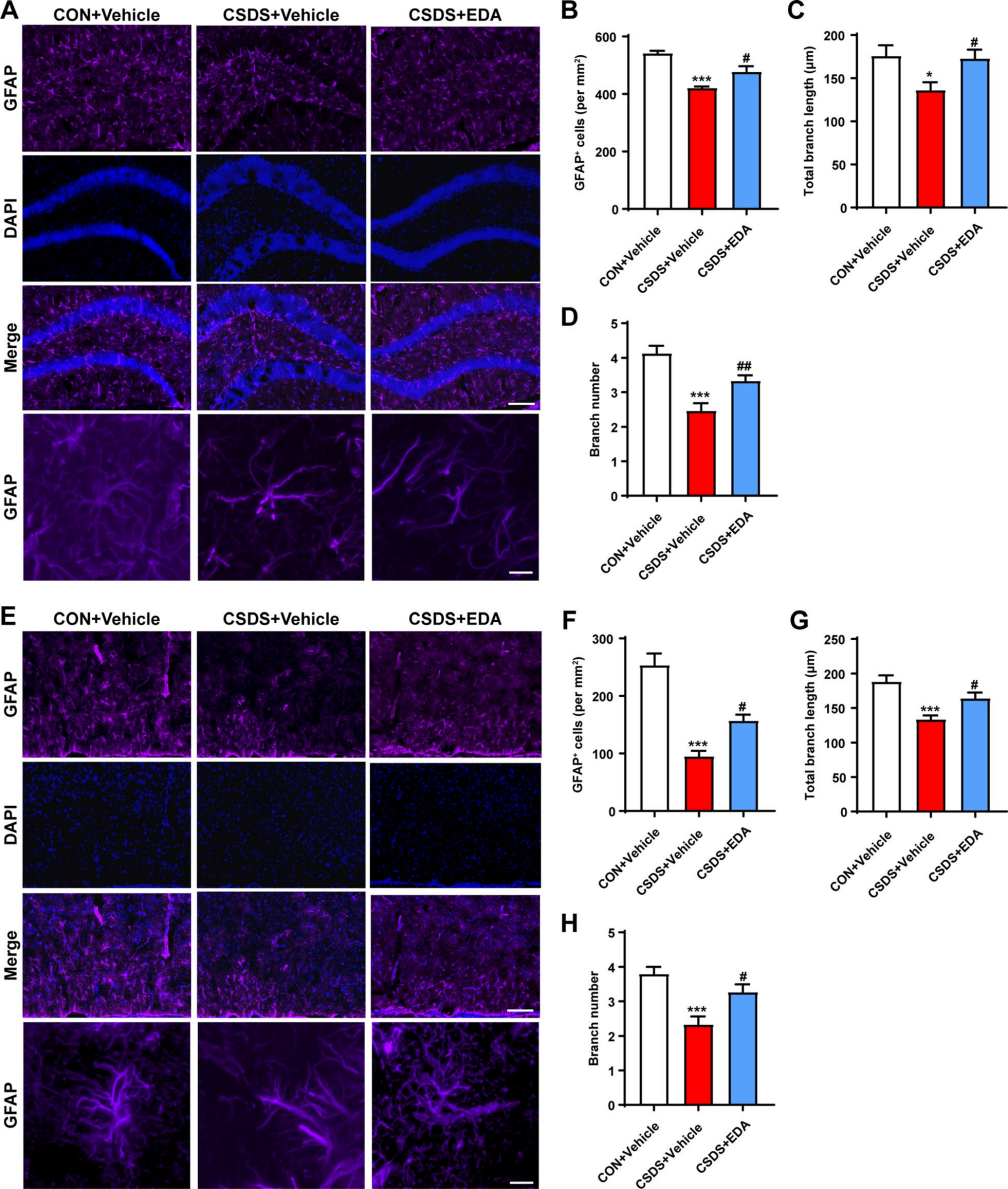
Effects of EDA on astrocyte loss in the CSDS mice Hip and mPFC. **A** Effect of EDA on astrocyte dysfunction-induced by CSDS in the Hip. Representative images of astrocyte immunostaining for GFAP. Scale bars, 100 μm (upper panel) and 10 μm (lower panel). **B** Quantification of GFAP-positive cells per mm^2^ in the Hip. **C** Total branch length. **D** Branch number. **E** Effect of EDA on astrocyte loss-induced by CSDS in the mPFC. Representative images of astrocyte immunostaining for GFAP. Scale bars, 100 μm (upper panel) and 10 μm (lower panel). **F** Quantification of GFAP-positive cells per mm^2^ in the Hip. **G** Total branch length. **H** Branch number. All the data are presented as mean ± SEM (*n* = 3 mice/group, 15 cells/group). *p < 0.05, ***p < 0.001 versus the CON + Vehicle group. ^#^*p* < 0.05, ^##^*p* < 0.01 versus the CSDS + Vehicle group

**Fig. 6:**
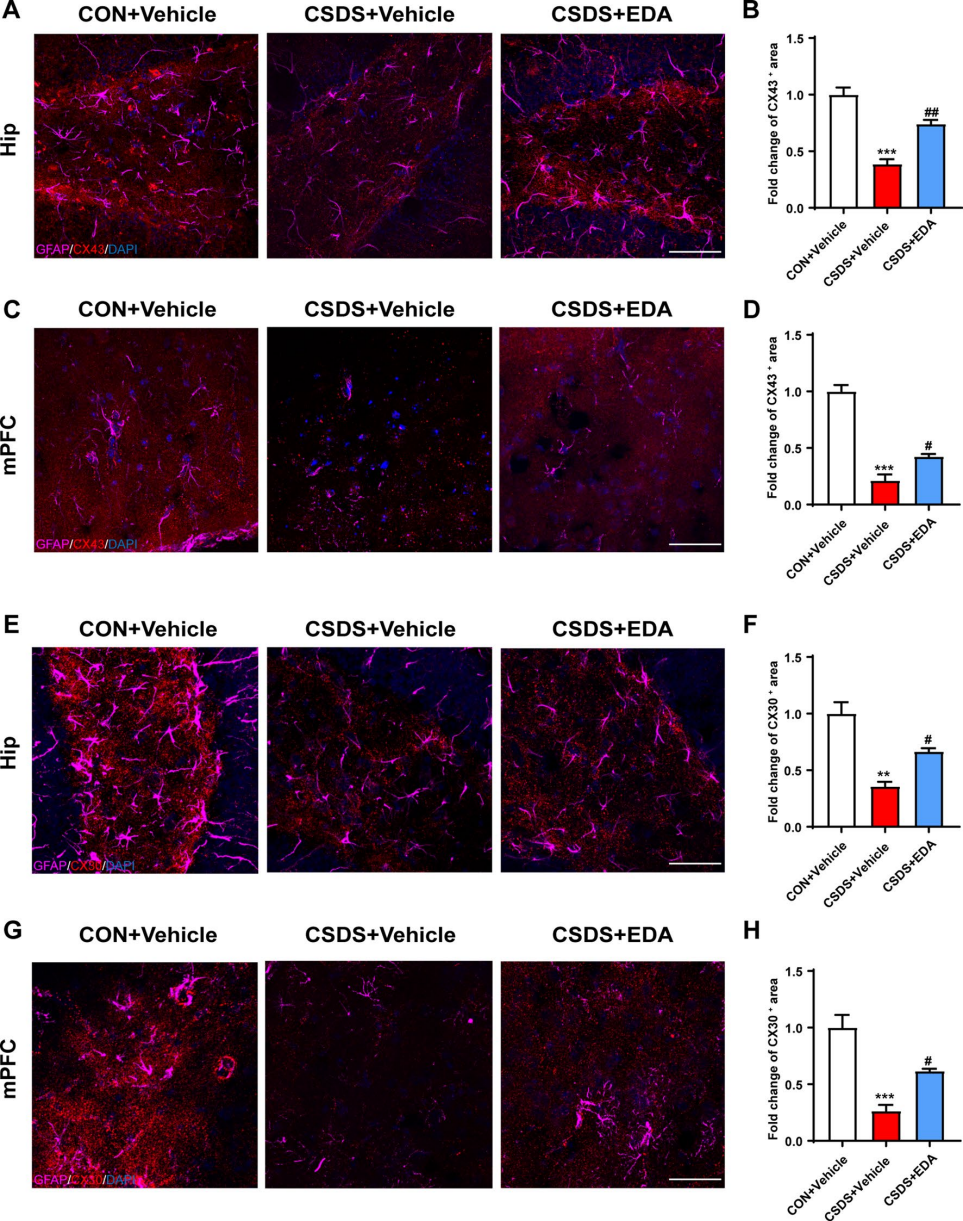
Administration of EDA increased the expression of astrocytic network proteins Cx43 and Cx30 in the Hip and mPFC. **A**, **B** Immunofluorescence staining of GFAP and Cx43 in the Hip. Scale bar, 50 μm. **C**, **D** Immunofluorescence staining of GFAP and Cx43 in the mPFC. Scale bar, 50 μm. **E**, **F** Immunofluorescence staining of GFAP and Cx30 in the Hip. Scale bar, 50 μm. **G, H** Immunofluorescence staining of GFAP and Cx30 in the mPFC. Scale bar, 50 μm. Data are presented as mean ± SEM (*n* = 3 per group). **p < 0.01, ***p < 0.001 versus the CON + Vehicle group. ^#^*p* < 0.05, ^##^*p* < 0.01 versus the CSDS + Vehicle group

The histological change was confirmed by glial fibrillary acidic protein (GFAP) staining (Fig. 5A, E). CSDS exposure resulted in astrocyte dysfunction in CSDS + Vehicle mice, as indicated by the fact that CSDS exposure decreased the number of GFAP-positive cells and by the ramification of astrocytes characterized by the significantly decreased branch numbers and length. As expected, administration of EDA remarkably alleviated astrocyte dysfunction (Fig. 5B–D, F–H). In addition, ELISA results also showed that CSDS stimuli dramatically decreased the protein level of GFAP in the Hip and mPFC (Fig. 4F, H). After EDA administration, the expression of GFAP was significantly increased in the Hip. There was an increased tendency in CSDS + EDA group as compared to CSDS + Vehicle group in the mPFC of GFAP. Mounting evidence certificated that astrocytic network proteins Cx43 and Cx30 are primarily expressed in astrocytes and help to regulate ion communication between astroglial activation and astroglial secretion [55, 56]. We observed down-regulation of astrocytic network markers Cx43 and Cx30 in CSDS exposed mice, while treatment with EDA significantly increased the expression of Cx43 and Cx30 in the Hip and mPFC (Fig. 6).Together, these results suggested that the microglial and astrocytic abnormality in CSDS exposed mice can be restored by the replenishment of EDA, suggesting a potential therapeutic strategy for depression.

### Effects of EDA on OS and mitochondrial damage in the Hip and mPFC

**Fig. 7:**
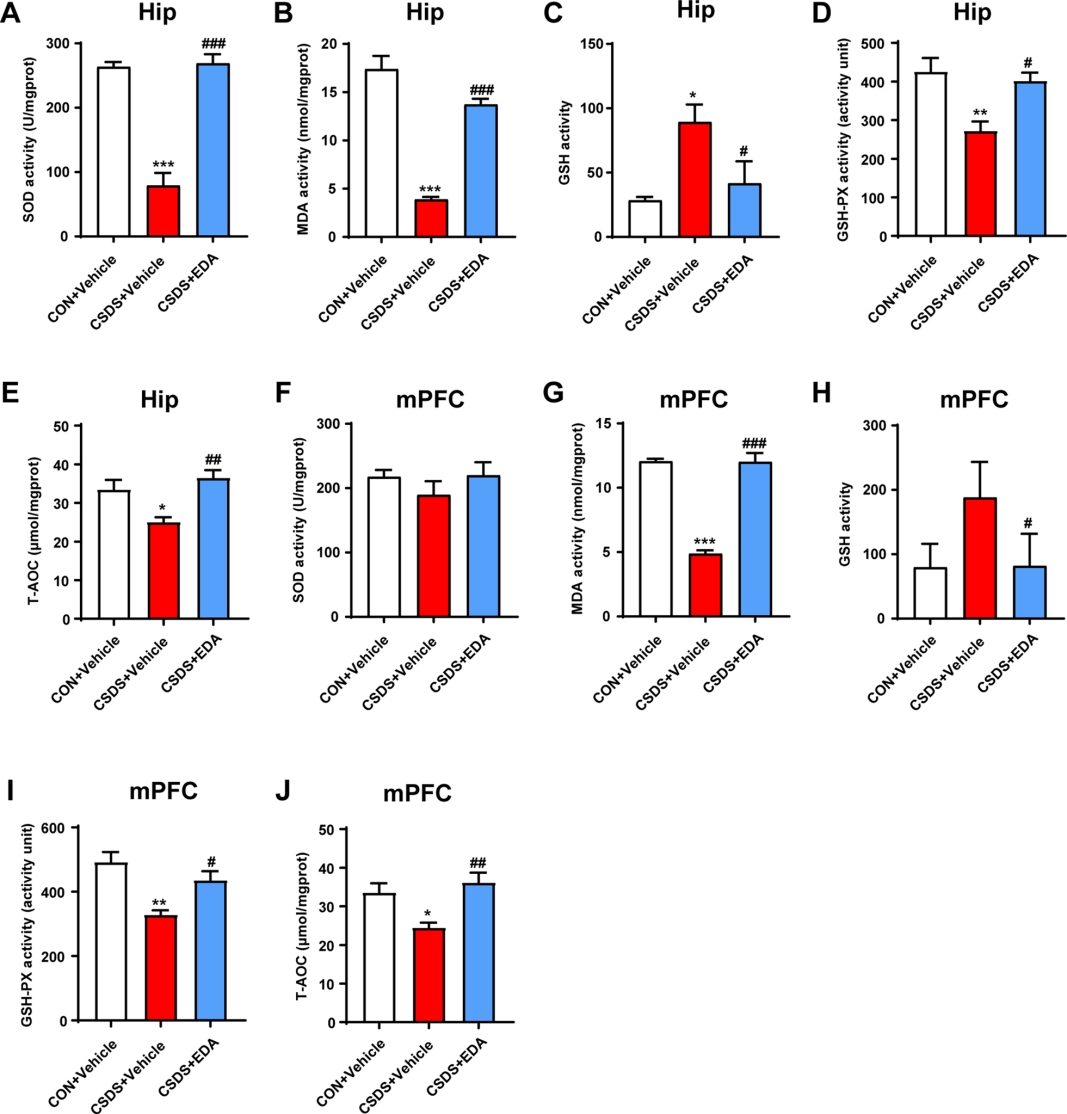
Changes of OS parameters in the Hip and mPFC after EDA exposure. **A**–**E** Levels of OS parameters including MDA, SOD,GSH,GSH-PX and T-AOC in the Hip. **F**–**J** MDA content, the activities of SOD, GSH, GSH-PX and T-AOC in the mPFC. Data are presented as mean ± SEM (*n* = 6 per group). *p < 0.05, **p < 0.01, ***p < 0.001 versus the CON + Vehicle group. ^#^*p* < 0.05, ^##^*p* < 0.01, ^###^*p* < 0.001 versus the CSDS + Vehicle group

Given OS is emerging as an important therapeutic target for MDD, we measured the activities of MDA, SOD, GSH, GSH-PX and T-AOC in the Hip and mPFC to evaluate the effect of EDA on OS induced lipid peroxidation. As shown in Fig. 7A, B, D, E, the activities of SOD, MDA, GSH-PX and T-AOC were significantly decreased in the Hip of CSDS + Vehicle mice. In contrast, the content of GSH in the CSDS + Vehicle group was notably higher than the CON + Vehicle group. However, intraperitoneal injection of EDA completely reversed these alterations (Fig. 7A–E). In the mPFC tissue, CSDS exposure significantly decreased MDA, GSH-PX and T-AOC levels. After EDA application, mice in the CSDS + EDA group recovered MDA, GSH, GSH-PX and T-AOC activities (Fig. 7G–J). Nevertheless, SOD activity had no changes among three groups (Fig. 7F).

**Fig. 8:**
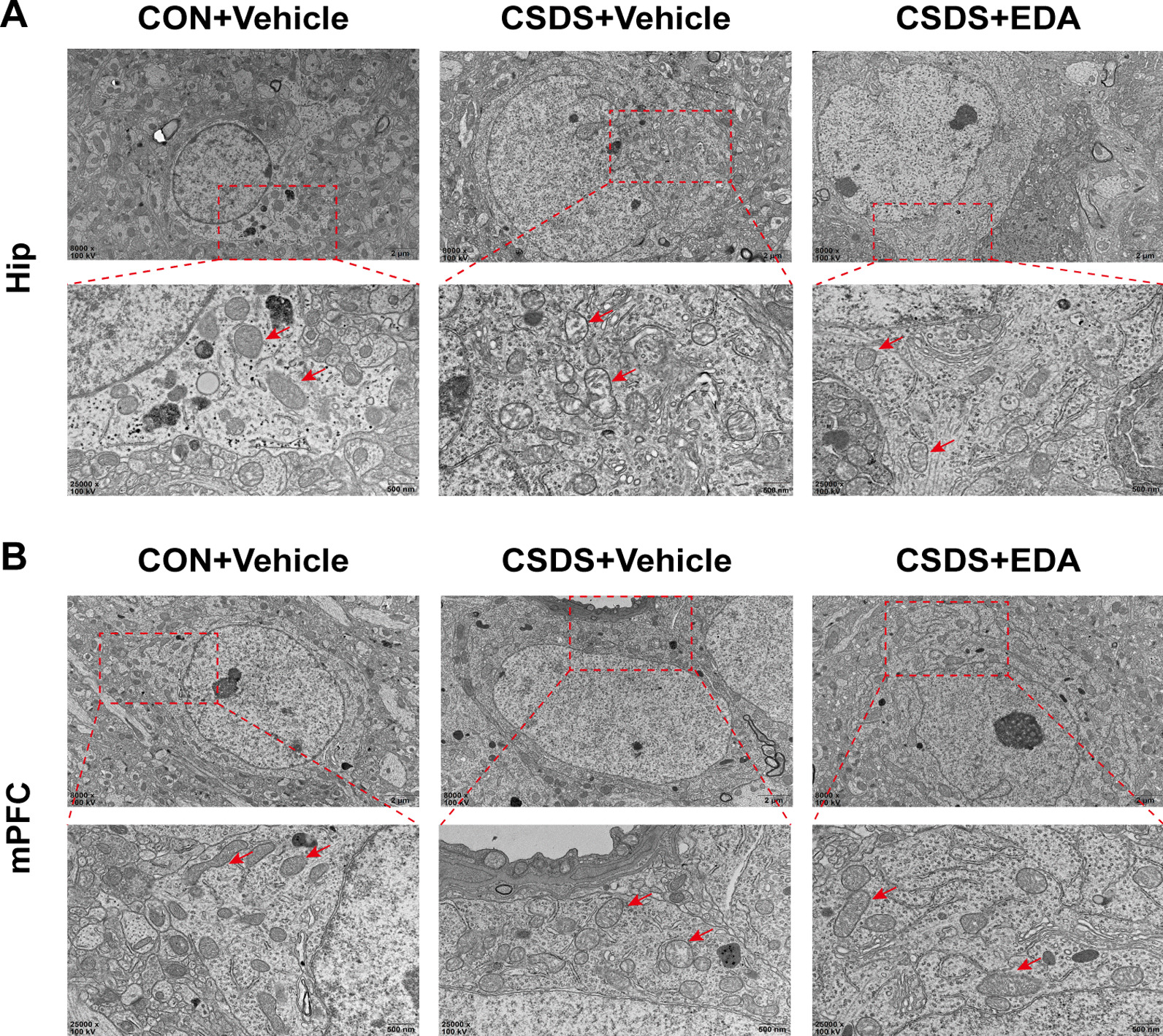
Ultrastructure of neurons in the CSDS model of Hip and mPFC with EDA treatment. **A** Electron micrographs showed mitochondrial damages in the hippocampal neuron (red arrows) Scale bars, 2 μm (upper panel) and 500 nm (lower panel). **B** Electron micrographs showed mitochondrial damages in the mPFC neuron (red arrows). Scale bars, 2 μm (upper panel) and 500 nm (lower panel)

Since the antioxidant property of EDA was mitochondria-mediated, we used TEM to observe mitochondrial damages in the Hip and mPFC. CSDS exposure exhibited profound effects on mitochondrial structure, including mitochondrial swelling and loss of mitochondrial cristae in neurons (Fig. 8) and microglia (Additional file 1: Fig. S1). In addition, compared with the CSDS + Vehicle group, EDA treatment alleviated these pathological changes. Interestingly, mitochondrial damage in the  Hip was more severe than in the mPFC region (Fig. 8A, B; Additional file 1: Fig. S1a, b). Altogether, these findings implied that EDA might ameliorate CSDS-induced OS and mitochondrial damage.

### EDA treatment affects energy metabolites in the Hip and mPFC

**Fig. 9:**
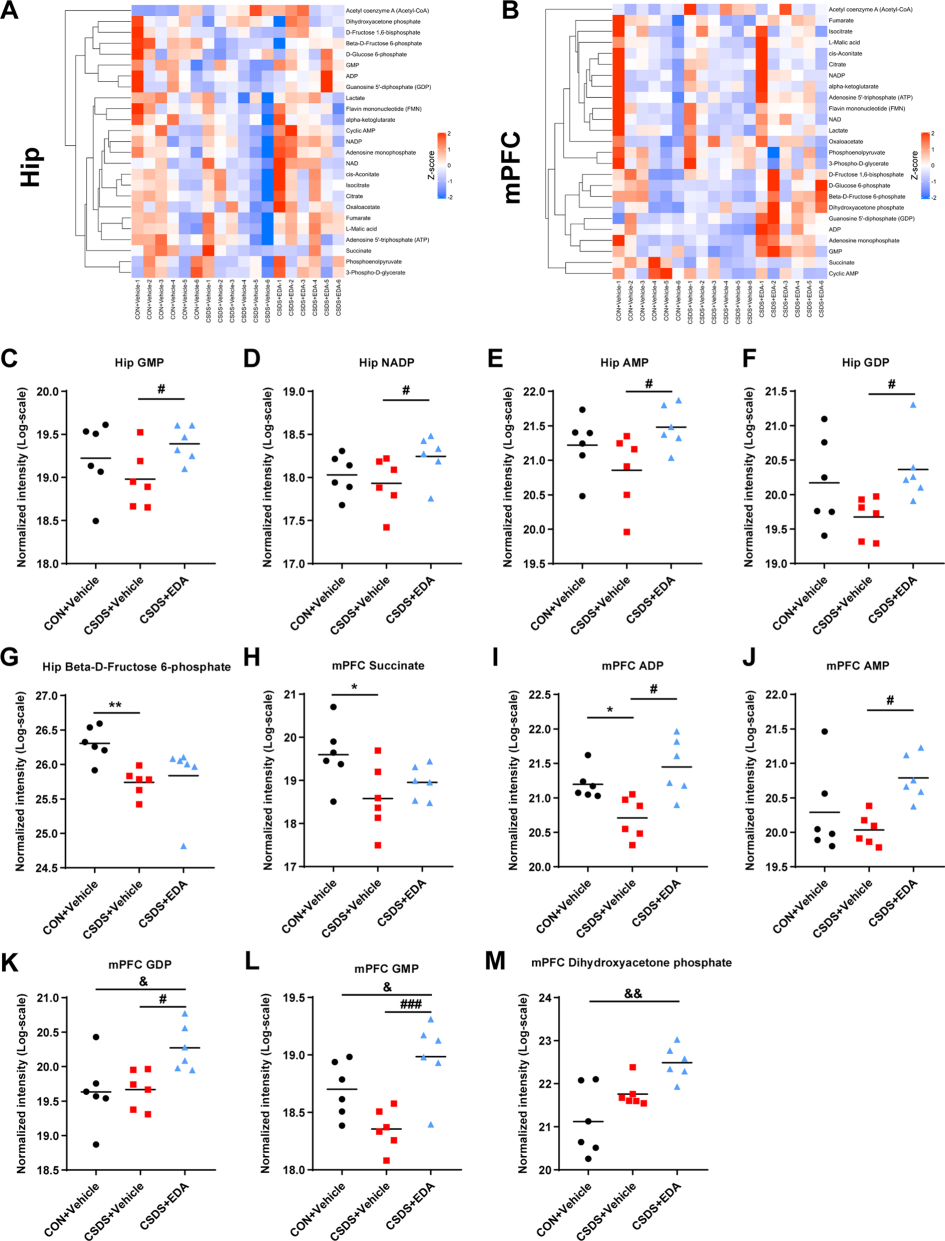
Targeted energy metabolomics in the Hip and mPFC of CSDS model mice. **A** Heatmap of partly energy-related metabolites in the Hip. **B** Heatmap of partly energy-related metabolites in the mPFC. Rows indicate metabolites. The degree of change in metabolite concentration is color coded. **C**–**G** Differential energy metabolites in the Hip. **H**–**M** Differential energy metabolites in the mPFC. Data are presented as mean ± SEM (*n* = 6 per group). *p < 0.05, **p < 0.01 versus the CON + Vehicle group. ^#^*p* < 0.05, ^###^*p* < 0.001 versus the CSDS + Vehicle group. ^&^*p* < 0.05, ^&&^*p* < 0.01 versus the CSDS + EDA group

Given EDA was a potent mitochondrial antioxidant and metabolism enhancer, we carried out targeted energy metabolomics analysis by detecting 32 metabolites in the Hip and mPFC tissues of mice. The heat maps of metabolites changes of CON + Vehicle versus CSDS + Vehicle versus CSDS + EDA are shown in Fig. 9A, B. In the Hip, the guanosine monophosphate (GMP), nicotinamide adenine dinucleotide phosphate (NADP), guanosine diphosphate (GDP) and adenosine monophosphate (AMP) in the CSDS + Vehicle group were reduced related to the CON + Vehicle group without statistical significance. However, these metabolites were significantly increased in the CSDS + EDA group compared with CSDS + Vehicle group (Fig. 9C–F). In addition, Beta-d-Fructose 6-phosphate in the CSDS + Vehicle group was notably diminished compared with CON + Vehicle group (Fig. 9G). In the mPFC, we found most of the energy-related metabolites were not changed by CSDS exposure. Nevertheless, Succinate and adenosine diphosphate (ADP) were significantly decreased in CSDS + Vehicle group compared with CON + Vehicle group (Fig. 9H, I). Meanwhile, compared with CSDS + Vehicle group, 4 metabolites (GMP, ADP, AMP and GDP) were markedly increased in the CSDS + EDA group (Fig. 9I–L). Among the differentially altered metabolites, GMP, GDP and AMP were altered in both the Hip and mPFC tissues, with the other metabolites altered either in Hip or mPFC.

Then, we used Ingenuity Pathway Analysis (IPA) to identify the top canonical signaling pathways affected in the Hip and mPFC with or without EDA treatment. Each of the top 5 canonical pathways was exhibited in Additional file 2: Fig. S2. Of all these pathways, we noticed that NAD salvage pathway II in the Hip was associated with the antioxidant mechanism of EDA. The NAD salvage pathway was the main source of NAD^+^ biosynthesis in mammalian cells [57]. Interestingly, Sirt1 is an NAD^+^ dependent histone deacetylase and mediates the levels of anxiety and depression [58].

Furthermore, we used Kyoto Encyclopedia of Genes and Genomes (KEGG) database to reveal the function of differential metabolites in signaling pathways. Remarkably, the significant alternation of NADP in the Hip might indicate a potential relevance between glutathione metabolism and depression.

Taken together, these results demonstrated that EDA administration significantly affected energy metabolites in the Hip and mPFC. Furthermore, changes in hippocampal energy metabolites might be more associated with depression in contrast with mPFC.

### EDA reverses Sirt1/Nrf2/HO-1/Gpx4 pathway and prevents CSDS-induced neuroinflammation

**Fig. 10:**
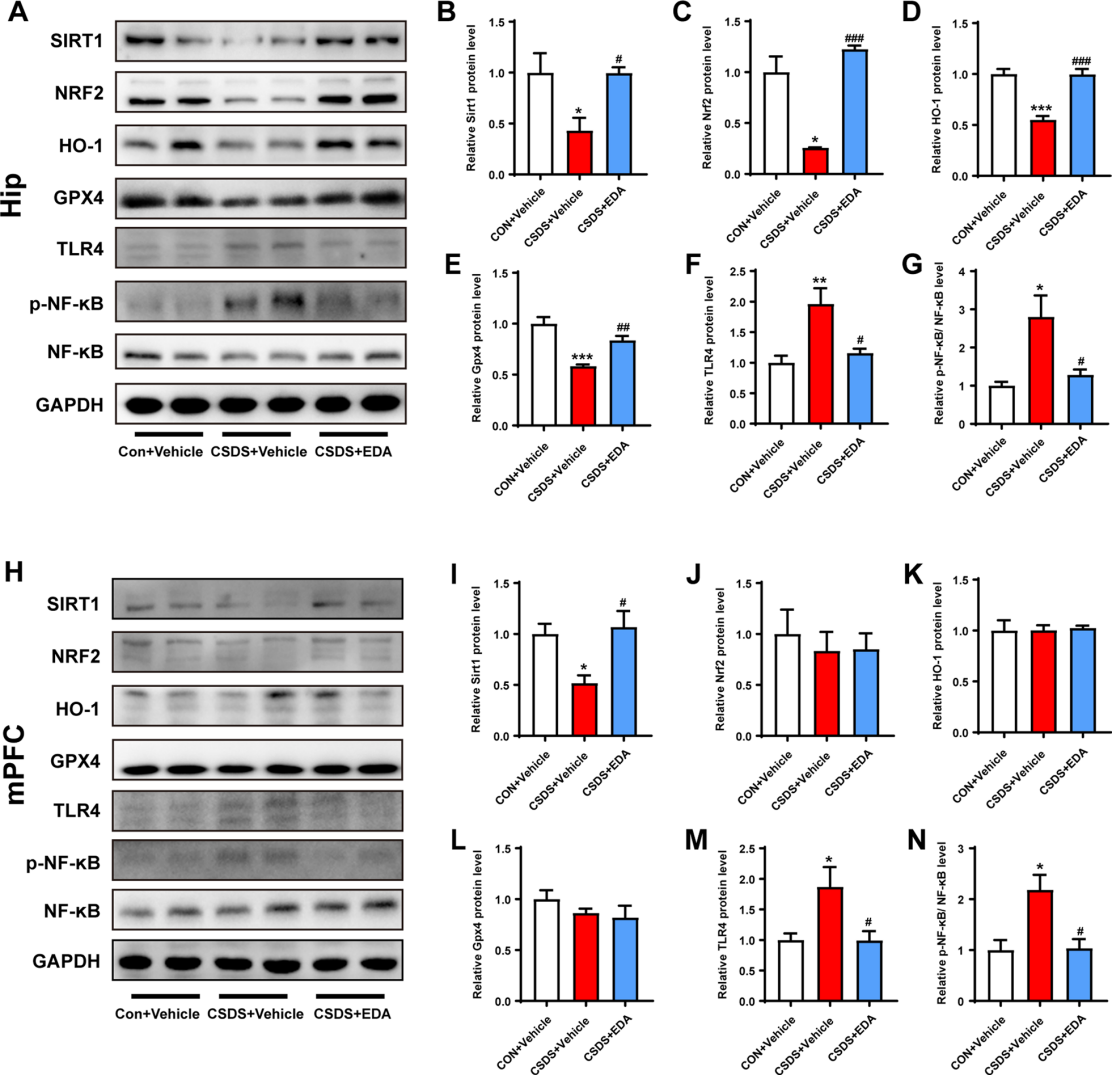
Effect of EDA on Sirt1/Nrf2/HO-1/Gpx4 and TLR4/NF-κB signaling pathways. **A** Representative WB bands in the hippocampal region. **B**–**G** Statistical graphs of relative protein expression of Sirt1 (**B**), Nrf2 (**C**), HO-1 (**D**), Gpx4 (**E**), TLR4 (**F**), and p-NF-κB (**G**). **H** Representative WB bands in the mPFC region. **I**–**N** Statistical graphs of relative protein expression of Sirt1 (**I**), Nrf2 (**J**), HO-1 (**K**), Gpx4 (**L**), TLR4 (**M**), and p-NF-κB (**N**). Data are presented as mean ± SEM (*n* = 4 per group). *p < 0.05, **p < 0.01, ***p < 0.001 versus the CON + Vehicle group. ^#^*p* < 0.05, ^##^*p* < 0.01, ^###^*p* < 0.001 versus the CSDS + Vehicle group

Given Gpx4 plays a crucial role in glutathione metabolism and the involvement of Sirt1 and Nrf2 in MDD, we next sought to examine the role of Sirt1/Nrf2/HO-1/Gpx4 pathway in EDA-related depression and anxiety by WB. As shown in Fig. 10A–G, CSDS exposure robustly decreased the expression levels of Sirt1, Nrf2, HO-1 and Gpx4 in the Hip. However, these effects were significantly reversed by the treatment with EDA. In the mPFC, the protein expression level of Sirt1 was significantly higher in CSDS + EDA group as compared to the CSDS + Vehicle group (Fig. 10I). Nevertheless, the CSDS + Vehicle and CSDS + EDA groups had no significant effects on the levels of Nrf2, HO-1 and Gpx4 (Fig. 10J–L).

**Fig. 11:**
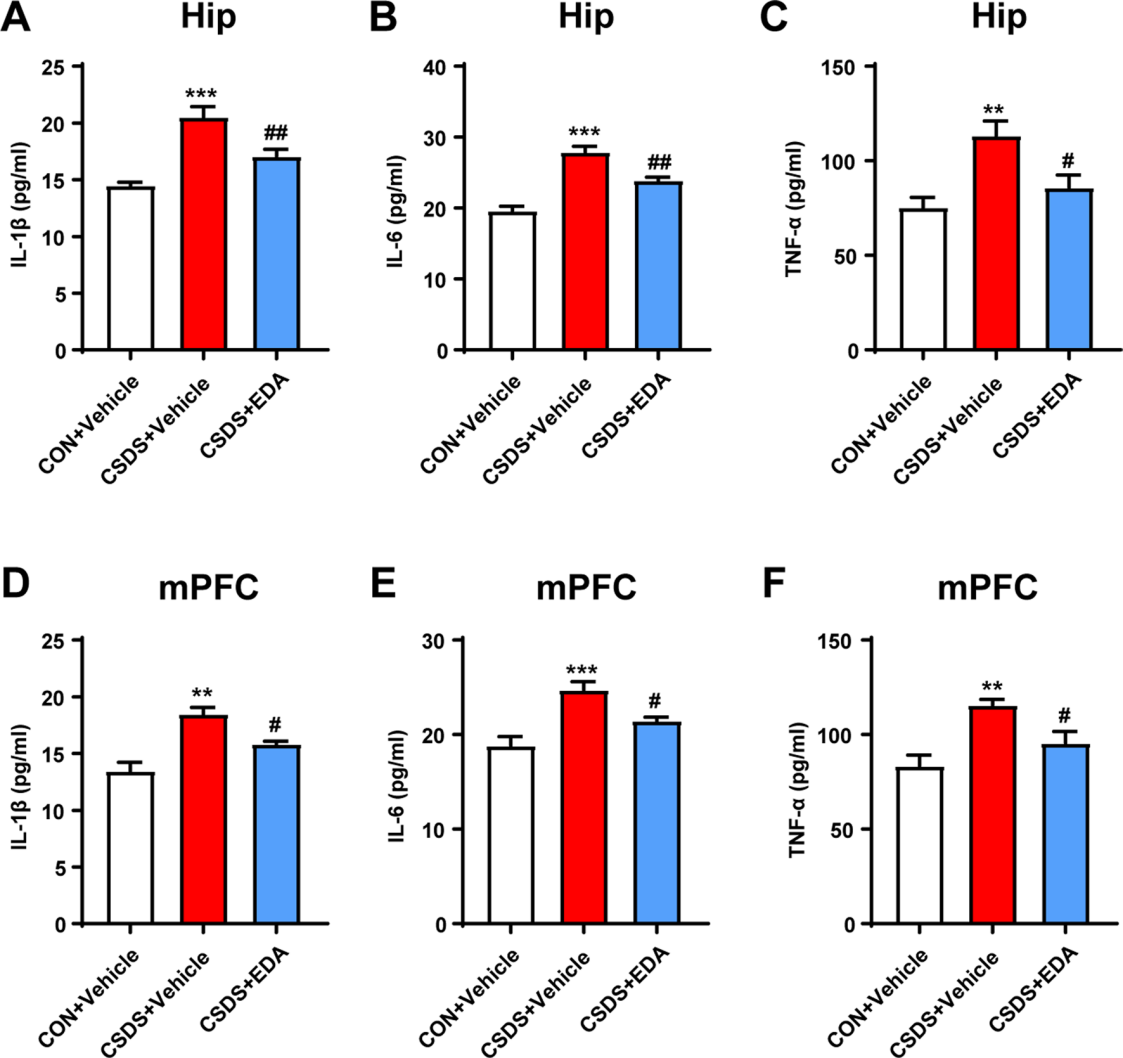
Effect of EDA on concentrations of IL-1β, IL-6, and TNF-α in the Hip and mPFC. **A**–**C** Concentrations of IL-1β (**A**), IL-6 (**B**), and TNF-α (**C**) in the hippocampal region. **D**–**F** Concentrations of IL-1β (**D**), IL-6 (**E**), and TNF-α (**F**) in the mPFC region. Data are presented as mean ± SEM (*n* = 6 per group). **p < 0.01, ***p < 0.001 versus the CON + Vehicle group. ^#^*p* < 0.05, ^##^*p* < 0.01 versus the CSDS + Vehicle group

Since TLR4/NF-κB signaling pathway mediated neuroinflammation plays a key role in neuropsychiatric disorders including MDD [12]. The expressions were detected by WB. As presented in Fig. 10A, F–H, M, N, the expression of TLR4 and p-NF-κB were dramatically increased following CSDS stimulation, which were all reversed by EDA administration. In addition, increased levels of IL-1β, IL-6 and TNF-α in the Hip and mPFC tissues induced by CSDS were remarkably suppressed by EDA injection (Fig. 11).

In order to evaluate Sirt1/Nrf2/HO-1/Gpx4 pathway in transcription level, we further examined the mRNA expressions of Sirt1, Nrf2, HO-1, and Gpx4 in the Hip and mPFC tissues. As exhibited in Additional file 3: Fig. S3, CSDS exposure did not change the expressions of Sirt1 in the Hip and Gpx4 in both regions. In addition, CSDS + Vehicle group was found with significantly up-regulated mRNA (Nrf2 and HO-1) in the Hip and mPFC as compared to CON + Vehicle group. Rather, within exposure to EDA, we found decreased expressions of Nrf2 and HO-1 in both Hip and mPFC compared with CSDS + Vehicle group.

Taken together, these results suggested that EDA reverses Sirt1/Nrf2/HO-1/Gpx4 pathway and abolishes CSDS-induced neuroinflammation.

### Blockade of Sirt1 abolishes the antidepressant effect of EDA via Sirt1/Nrf2/HO-1/Gpx4 pathway

**Fig. 12:**
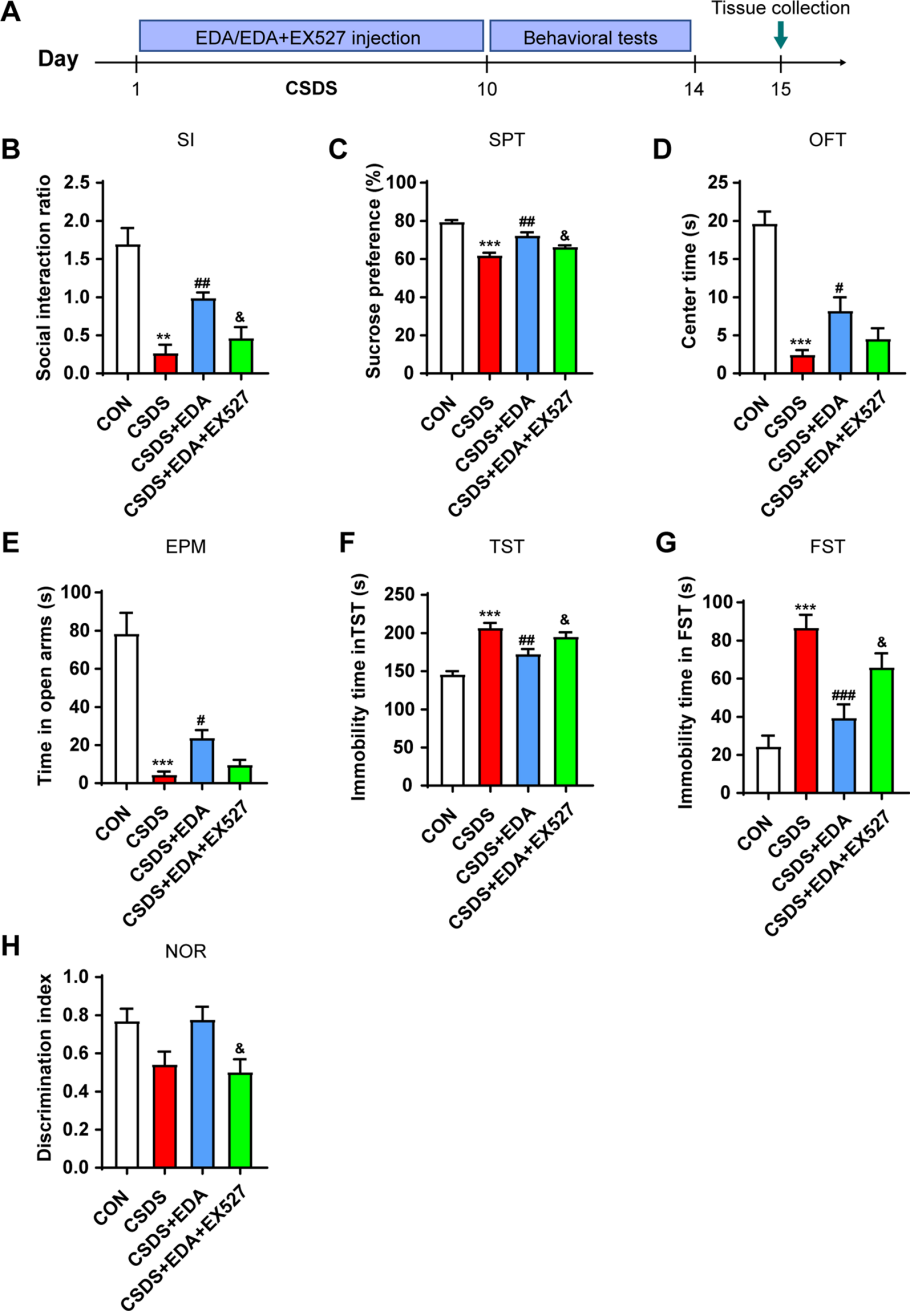
Blockade of Sirt1 abolished the antidepressant and anxiolytic effects of EDA. **A** Schematic timeline of the experimental procedure. **B** Social interaction test. **C** Sucrose preference test. **D** Open field test. **E** Elevated plus maze test. **F** Tail suspension test. **G** Forced swimming test. **H** Novel object recognition test. All the data are expressed as mean ± SEM (*n* = 8 per group). **p < 0.01, ***p < 0.001 versus the CON group. ^#^*p* < 0.05, ^##^*p* < 0.01, ^###^*p* < 0.001 versus the CSDS group. ^&^*p* < 0.05 versus the CSDS + EDA group

To investigate the effects of Sirt1 on the antidepressant and anxiolytic effects of EDA, EX527 (a Sirt1 inhibitor) was administered in CSDS-induced mice 30 min before the administration of EDA (Fig. 12A). As shown in Fig. 12B–E, H, compared with the CSDS group, SI ratio, sucrose consumption, center time and open arms time increased significantly after the administration of EDA. However, when EDA and EX527 were administered together, EX527 notably reduced the EDA- induced increase in SI ratio, sucrose consumption and discrimination index, but not in center time and open arms time. In Fig. 12F, G, treatment with EX527 increased immobility time in TST and FST compared with those in CSDS + EDA group.

**Fig. 13:**
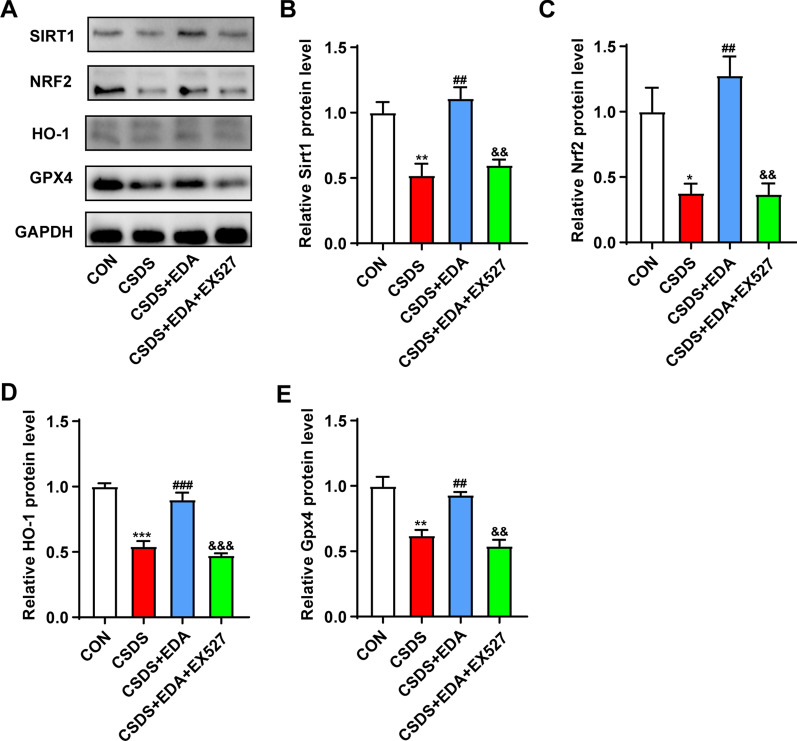
Impact of Sirt1 inhibitor on EDA-induced Sirt1/Nrf2/HO-1/Gpx4 signaling pathway in the Hip of CSDS mice. **A** Representative WB bands. **B**–**E** Protein levels of Sirt1 (**B**), Nrf2 (**c**), HO-1 (**D**), and Gpx4 (**E**) in the Hip. Data are presented as mean ± SEM (*n* = 3 per group). *p < 0.05, **p < 0.01, ***p < 0.001 versus the CON group. ^##^*p* < 0.01, ^###^*p* < 0.001 versus the CSDS group. ^&&^*p* < 0.01, ^&&&^*p* < 0.001 versus the CSDS + EDA group

To further validate the role of Sirt1in EDA-mediated antidepressant effect, the proteins in the Hip, including Sirt1, Nrf2, HO-1 and Gpx4 level were measured using WB. As shown in Fig. 13, the expression of Sirt1, Nrf2, HO-1 and Gpx4 was significantly higher in CSDS + EDA mice as compared to the CSDS  group. However, inhibition of Sirt1 with EX527 abolished the effects of EDA, which led to a decrease in Sirt1, Nrf2, HO-1 and Gpx4 levels as compared to the CSDS + EDA group.

Taken together, these results suggested that inhibition of Sirt1 could suppress the antidepressant effect of EDA via Sirt1/Nrf2/HO-1/Gpx4 pathway.

### Blockade of Nrf2 abolishes the antidepressant and anxiolytic effects of EDA via Sirt1/Nrf2/HO-1/Gpx4 pathway

**Fig. 14:**
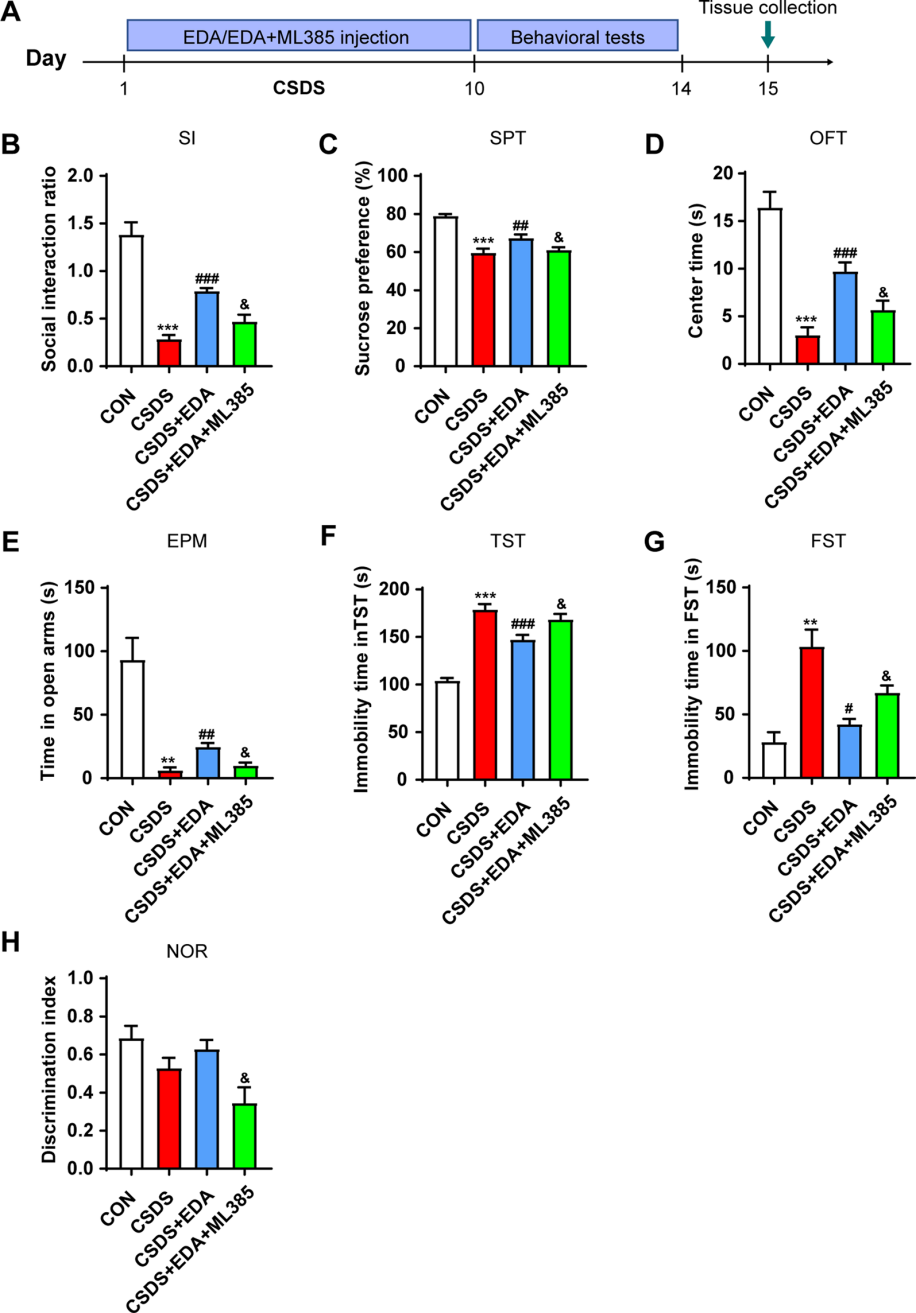
Blockade of Nrf2 abolished the antidepressant and anxiolytic effects of EDA. **A** Schematic timeline of the experimental procedure. **B** Social interaction test. **C** Sucrose preference test. **D** Open field test. **E** Elevated plus maze test. **F** Tail suspension test. **G** Forced swimming test. **H** Novel object recognition test. All the data are expressed as mean ± SEM (*n* = 8 per group). **p < 0.01, ***p < 0.001 versus the CON group. ^#^*p* < 0.05, ^##^*p* < 0.01, ^###^*p* < 0.001 versus the CSDS group. ^&^*p* < 0.05 versus the CSDS + EDA group

**Fig. 15:**
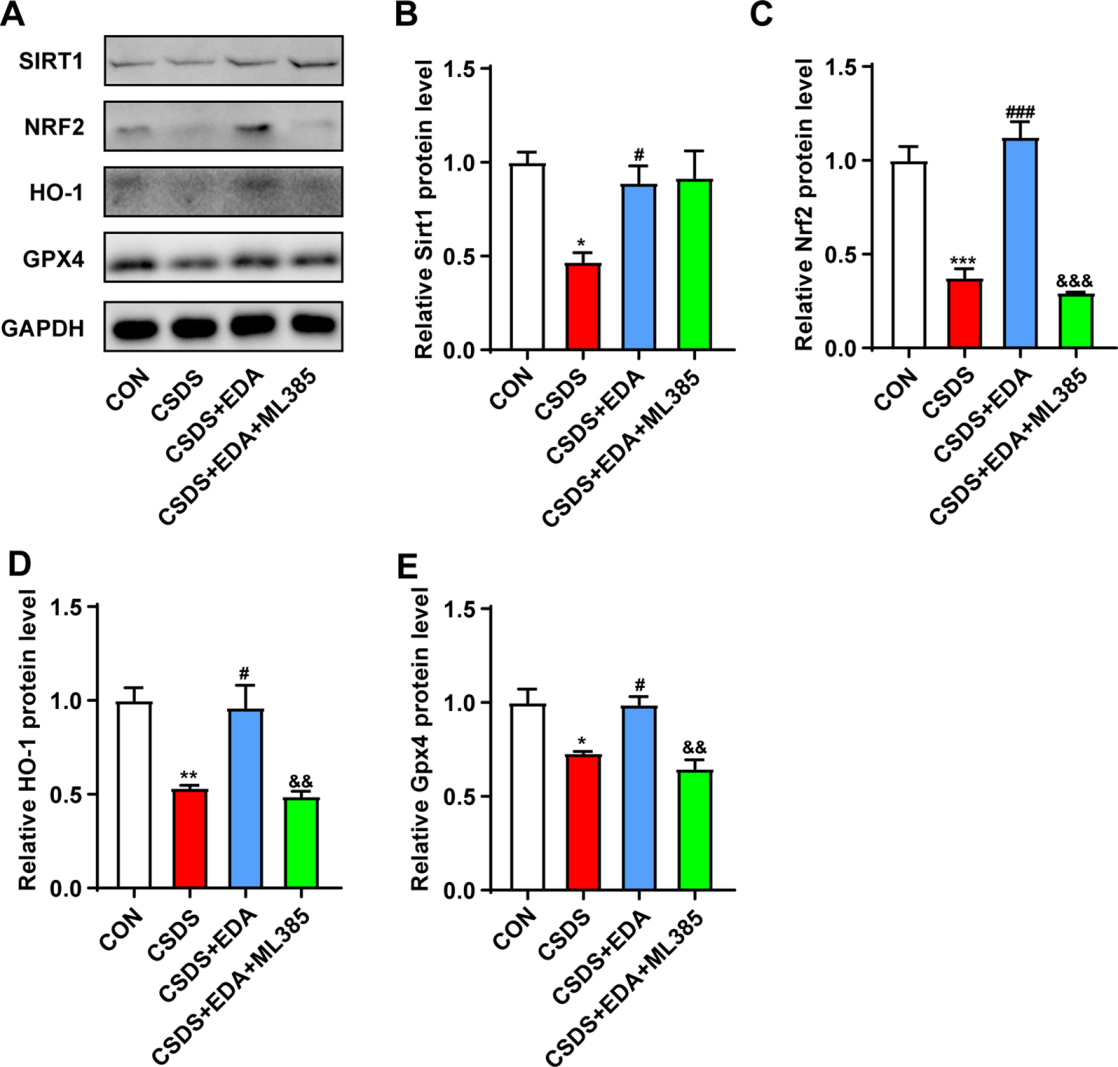
Impact of Nrf2 inhibitor on EDA-induced Sirt1/Nrf2/HO-1/Gpx4 signaling pathway in the Hip of CSDS mice. **A** Representative WB bands. **B**–**E** Protein levels of Sirt1 (**B**), Nrf2 (**C**), HO-1 (**D**), and Gpx4 (**E**) in the Hip. Data are presented as mean ± SEM (*n* = 3 per group). *p < 0.05, **p < 0.01, ***p < 0.001 versus the CON group. ^#^*p* < 0.05, ^###^*p* < 0.001 versus the CSDS group. ^&&^*p* < 0.01, ^&&&^*p* < 0.001 versus the CSDS + EDA group

To further evaluate whether Nrf2 is a downstream target of Sirt1, mice were treated with Nrf2 inhibitor ML385 (Fig. 14A). Treatment with EDA increased SI ratio, sucrose consumption, center time and open arms time and decreased immobility time in TST and FST compared with those in the CSDS  group. On the other hand, compared to CSDS + EDA group, the ML385 treatment markedly decreased SI ratio, sucrose consumption, center time, open arms time and discrimination index and increased immobility time in TST and FST (Fig. 14B–H). Next, we explored Sirt1, Nrf2, HO-1 and Gpx4 expressions in the Hip after ML385 treatment. As described in Fig. 15, in response to CSDS exposure, we observed a decrease in the protein levels of Sirt1, Nrf2, HO-1 and Gpx4. Treatment with EDA resulted in a significant up-regulation of those proteins. However, application of ML385 abolished the effects of EDA on Nrf2, HO-1 and Gpx4, while it had no significant effect on the expression of Sirt1. These results confirmed that Nrf2 is the downstream target of Sirt1.

### Blockade of Gpx4 abolishes the antidepressant and anxiolytic effects of EDA

**Fig. 16:**
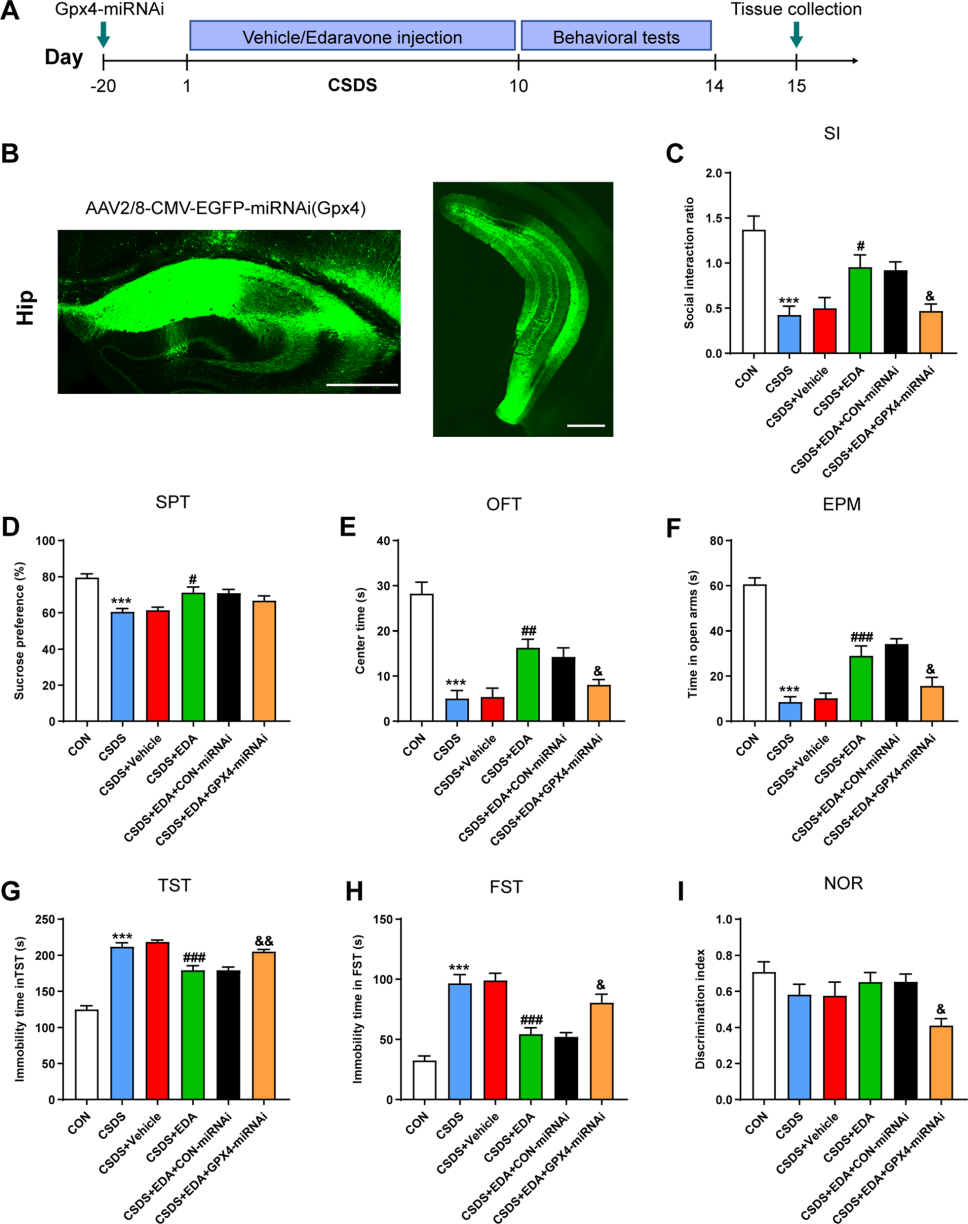
Knockdown Gpx4 in the Hip antagonized the antidepressant and anxiolytic effects of EDA. **A** Schematic timeline of the experimental procedure. **B** Fluorescence image of a fixed brain section that expressed AAV2/8-CMV–EGFP–miRNAi(Gpx4) in the Hip. Scale bar, 500 μm. **C** Social interaction test. **D** Sucrose preference test. **E** Open field test. **F** Elevated plus maze test. **G** Tail suspension test. **H** Forced swimming test. **I** Novel object recognition test. All the data are expressed as mean ± SEM (*n* = 10 per group). ***p < 0.001 versus the CON group. ^#^*p* < 0.05, ^##^*p* < 0.01, ^###^*p* < 0.001 versus the CSDS group. ^&^*p* < 0.05, ^&&^*p* < 0.01 versus the CSDS + EDA group

Previous studies and our results have confirmed that Sirt1 and Nrf2 played an important role in depression [24, 59]. It remains unknown, however, whether Gpx4-regulated ferroptosis affect the antidepressant and anxiolytic effects of EDA. Accordingly, an adeno-associated virus (AAV) vector that selectively expresses Gpx4–miRNAi with enhanced green fluorescent protein (AAV–EGFP–Gpx4–miRNAi) was injected into the Hip of mice. Then CSDS depression model was performed, followed by behavior tests by SI, SPT, OFT, NOR, EPM, TST, and FST (Fig. 16A, B). As shown in Fig. 16C–F, mice exposed to EDA showed markedly increased SI ratio in SI, sucrose consumption in SPT, center time in OFT and open arms time in EPM as compared to the CSDS group, whereas compared to the CSDS + EDA group, the AAV–EGFP–Gpx4–miRNAi treatment decreased SI ratio, center time and open arms time, but not sucrose consumption. As shown in Fig. 16G, H, immobility time in TST and FST was significantly decreased in the CSDS + EDA group as compared to the CSDS group. However, compared to the CSDS + EDA group, immobility time in TST and FST in the CSDS + EDA + Gpx4–miRNAi group was dramatically increased. Intriguingly, the discrimination index exhibited markedly decreased in the CSDS + EDA + Gpx4–miRNAi group as compared to the CSDS + EDA group (Fig. 16I). These data indicated that the antidepressant and anxiolytic effects of EDA in CSDS-induced mice were blocked by AAV–EGFP–Gpx4–miRNAi.

## Discussion

**Fig. 17:**
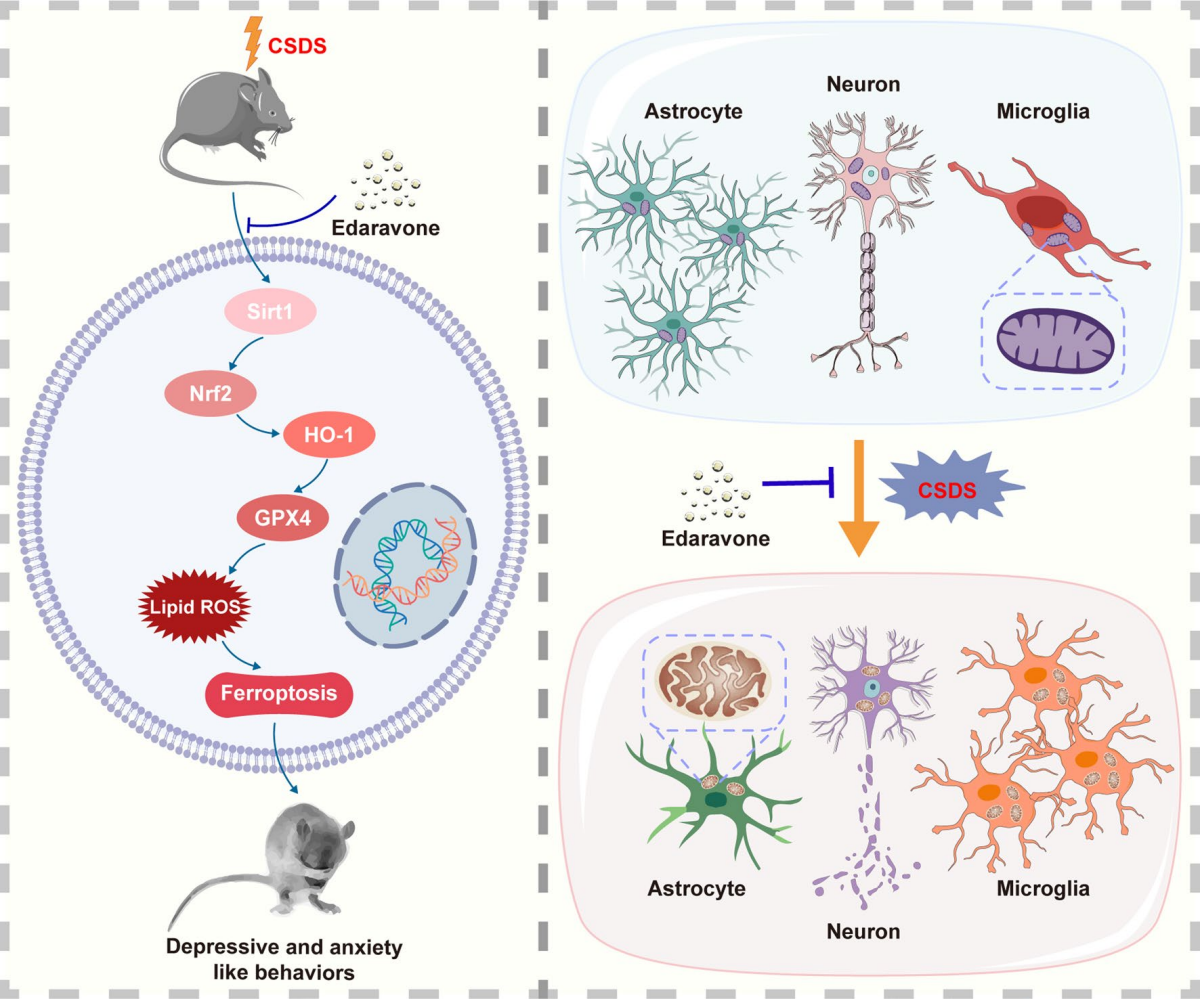
Schematic illustration of the proposed mechanisms underlying EDA-related antidepressant and anxiolytic efficacy

In the present study, we demonstrated that the antidepressant and anxiolytic properties of EDA and its potential mechanisms (Fig. 17). Current studies indicated that EDA attenuated CSDS-evoked depressive symptoms and CSDS-induced anxiety behaviors, and the underlying mechanisms were associated with the alteration of expression of Sirt1/Nrf2/HO-1/Gpx4 pathway in the Hip. The knockdown of Gpx4 in the Hip abolished the effects of EDA treatment. Furthermore, EDA protected microglia and astrocyte against CSDS-induced inflammation alternations, attenuated neuronal damage and pro-inflammatory cytokines activation in the Hip and mPFC. These results shed light on the antidepressant and anxiolytic effects of EDA and provide a new idea about the Gpx4-regulated ferroptosis in depression and anxiety.

To the best of our knowledge, this study is the first to demonstrate that the antidepressant and anxiolytic effects of EDA were brain region-specific. EDA is a free radical scavenger that can pass through the blood-brain barrier (BBB) and has therapeutic effects on stroke and ALS [35]. Only a few studies indicated that high concentrations of EDA presented antidepressant-like activity in chronic restraint stress and corticosterone model of depression [37, 38]. Here, we first evaluated the effect of EDA on CSDS-induced depressive behaviors. As expected, administration of EDA ameliorated depressive and anxiety-like behaviors.

Recent studies confirmed that MDD was associated with hippocampal and mPFC structural aberrations, including cellular damage, volumetric reductions and reduced hippocampal neurogenesis [60, 61]. Thus, we focused on Hip and mPFC regions and sought to explore whether the antidepressant and anxiolytic effects of EDA were present in specific region of the brain. Here, we found that the anti-neuroinflammation effect of EDA in CSDS-induced depressed mice was associated with inhibiting the increase of microglial activation and mitigating astrocyte dysfunction in the Hip and mPFC regions. In addition, the neuronal death and pro-inflammatory cytokines activation in the Hip and mPFC were relieved with treatment of EDA. MDD was initially speculated to be a disease derived from neuronal dysfunction [49]. However, numerous studies had linked astrocyte dysfunction to the pathophysiology of MDD [62]. In rodent depression models or postmortem patients with MDD patients, the expression of GFAP reduced in the Hip and prefrontal cortex as compared to controls. The reason for reduced astrocytes in CSDS mice may be related to potassium imbalance. Chronic stress, leading to depression, can up-regulate the inwardly rectifying potassium channel, K_ir_ 4.1 expression in astrocytic membrane processes that wrap tightly around the neuronal soma [63]. This channel on astrocytes tightly regulates the degree of membrane hyperpolarization and burst firing of neurons, which may implicate astrocytes with depression. Then, astrocytes are lost. This will destabilize glutamate homeostasis by reducing glutamate reuptake and synthesis. Furthermore, the decrease in astrocyte densities is also related to decreased expression of CX30 [64].

OS and inflammation are interdependent and functionally complementary, which are ubiquitous in MDD. Mounting evidence indicated that inflammation may affect mitochondrial function, membrane polarity and oxidative phosphorylation, which may further lead to OS and apoptosis [11, 65–67]. Microglia and astrocytes are main mediators of inflammation in the brain [68]. In our study reported here, the EDA administration significantly decreased microglial activation and attenuated astrocyte dysfunction. In addition, our results were consistent with previous studies showing that the chronic stress exposure decreased the activities of SOD, GSH-PX and T-AOC and increased GSH activity, indicating a direct involvement of OS in depression [69]. However, treatment with EDA alleviated this damage. Ferroptosis is oxidative damage-related regulated cell death, mainly driven by iron accumulation, lipid peroxidation, and subsequent plasma membrane rupture [70]. Surprisingly, compared with previous studies that showed elevated MDA activity in depression, our results showed that MDA activity was lower in CSDS model mice compared to controls. Some studies also reported divergent results on MDA level. Dionisie et al. showed that there was no significant increase in the MDA level of frontal cortex under chronic unpredictable mild stress (CUMS) model exposed rats [71]. Similarly, Wang et al. also demonstrated that the level of MDA in the Hip and cortex did not change in the CUMS-induced rats as compared to controls [72]. Bai et al. reported that the MDA level slightly reduced in the serum of schizophrenia patients compared with controls [73]. Decreased MDA level has also been reported in other diseases. Ma et al. showed a decrease of serum MDA level in gastric cancer patients compared with those in control subjects [74]. Punnonen et al. found decreased tissue MDA level in human breast cancer [75]. Similarly, Chang and Biasi et al. showed decreased serum MDA level in colon cancer [76, 77]. Murugan et al. showed significantly decreased serum MDA level in gastric carcinogenesis in rat model [78].

However, the reason for the discrepancy about MDA is elusive. Mounting studies showed that lipid peroxidation plays a central role in mediating ferroptosis. MDA, 4-hydroxynonenal (4-HNE) and 8-hydroxydeoxyguanosine (8-OH-dG), the final products of lipid peroxidation, have been applied in various samples in vitro and in vivo [79]. The MDA assay kits we used started with its measurement a component of thiobarbituric acid-reactive substances (TBARS) for the assessment of lipid peroxidation by spectrophotometry or fluorimetry. However, this method lacks specificity, because compounds containing many chemically reactive carbonyl groups in different types of substances can react with thiobarbituric acid (TBA) and interfere with MDA analysis [80]. These reactive carbonyl-containing compounds include oxidized polyunsaturated fatty acids and carbohydrates from endogenous sources and food in body fluids. Therefore, the specificity and sensitivity using MDA assay kit are controversial, which may affect the interpretation of the results. Therefore, using TBA as a sole indicator of lipid peroxidation is not appropriate.

Increasing evidence indicated that mitochondrial dysfunctions have been deemed as the underlying mechanism of MDD and the increase of ROS is associated with the reduction in neuronal metabolism [81, 82]. This metabolic deterioration is associated with reduced activity of ATP synthesis, primarily due to mitochondrial dysfunction. According to the “mitochondrial bioenergetics hypothesis”, depression is the most common psychiatric disorder in patients with impaired mitochondrial functions [82]. Given the most prominent role of mitochondria is the production of energy, we then performed TEM and targeted energy metabolomics to further explore the mitochondrial dysfunctions and abnormal energy metabolism in the Hip and mPFC tissues of depression. Previous works have shown significant disturbances of energy metabolism in CSDS, CUMS, learned helplessness, and chronic restraint stress model of mice [45, 83]. In our study, intraperitoneal injection of EDA greatly improved the mitochondrial and energy metabolism dysfunctions. Of note, mitochondrial damage in the Hip was more severe than in the mPFC region.

The changes of energy metabolism pathway are involved in the Krebs cycle and glycolysis. We then used IPA and KEGG to analyze the differentially expressed metabolites. It is worth noting that NAD salvage pathway II through IPA and glutathione metabolism via KEGG pathway enrichment in the Hip might be associated with the antioxidant mechanism of EDA. Sirt1, a NAD^+^-dependent deacetylase, plays a key role in NAD salvage pathway II and mediates the levels of anxiety and depression [58, 59]. Sirt1 expression was markedly reduced in the blood of MDD patients compared with healthy subjects [84]. Moreover, the Sirt1 activity in dentate gyrus (DG) of Hip was decreased when chronic stress exposure and genetic or pharmacologic ablation of Hip Sirt1 can lead to depression [85]. Nrf2, an important antioxidant, is an important downstream target of Sirt1 and plays a crucial role in improving the resistance to oxidative stress damage. Previous studies showed that Nrf2 knockout mice exhibited depressive-like behavior and CUMS model decreased Nrf2 expression in the rat Hip [86, 87]. Besides, corticosterone treated mice showed a lower level of Nrf2 protein expression in the cortex and Hip [88]. Recently, accumulating evidence revealed that a close link between Nrf2/HO-1 pathway and MDD [88, 89]. It is mentioned above that we found the difference in glutathione metabolism via KEGG pathway enrichment. Interestingly, the gene of Gpx4 plays a key role in glutathione metabolism and Nrf2 can directly or indirectly regulate Gpx4 protein expression and function [90].

Therefore, we speculated that EDA improved depressive and anxiety-like behaviors by activating the Sirt1/Nrf2/HO-1/Gpx4 signaling pathway. Here, we observed that mice underwent CSDS exposure displayed reduced protein expressions of Sirt1, Nrf2, HO-1 and Gpx4 in the Hip and Sirt1 in the mPFC, which were all elevated by EDA intervention. The reasons for the divergent results between the Hip and mPFC are unclear, but one possibility may be due to different neurons having different levels of vulnerability to OS [91]. Previous studies reported that Hip was the most susceptible region to OS. It is known that the dentate gyrus (DG–cornu ammonis 3(CA3) showed structural plasticity. Mounting studies showed that pyramidal cells in CA3 and granule cells in DG are OS prone regions, whereas other literature reported that pyramidal neurons in CA1 are more susceptible to oxidative stress. It was reported that oxidative damage of DG-CA1/3 function can reduce neuron proliferation, alter neuronal plasticity and disrupt neurogenesis. On the other hand, the frontal cortex seems to be more resilient to OS [71]. Chronic stress stimuli may cause dendritic shrinking and alter neuronal connectivity within mPFC [91].

Interestingly, mRNA levels of Nrf2, HO-1 in the Hip and mPFC and Sirt1 in the mPFC increased, while its protein levels in the Hip were down-regulated. We deduced this disparity between mRNA and protein levels suggests that post-transcriptional regulation, translational efficiency, and post-translational modifications alter protein levels. One possible explanation is that reduced translational efficiency may be compensated by increased transcriptional activity [92]. However, the detailed mechanism is not clear.

Ferroptosis is a recently identified type of regulated cell death and has also been reported to be associated with several neurological diseases, including neurodegeneration, stoke and neurotrauma [93]. However, there is still lack of reports about ferroptosis in the psychiatric diseases, especially depression and anxiety. First, we employed TEM to examine the ultrastructure of neurons and microglia in the Hip and mPFC. Surprisingly, we found the most prominent characteristic of ferroptosis, reduction volume, ruptured mitochondria in the hippocampal microglia of CSDS exposed mice. Consistent with the shrunken mitochondria observed in hippocampal microglia, CSDS-induced mice had lower SOD, GSH-PX and T-AOC levels, as well as higher GSH level in the hippocampal tissue. Furthermore, our results showed that the expression of ferroptosis-related proteins, including Gpx4 and Nrf2 were down-regulated in the Hip of CSDS exposed mice. Intriguingly, these effects were all reversed with EDA treatment. Taken together, these results inferred that ferroptosis could play a role in depression and anxiety and EDA can relieve CSDS-induced ferroptosis.

Since Gpx4 was identified as a key regulatory factor in ferroptosis and the protein expression of Gpx4 changed after CSDS exposure in the Hip, but not in the mPFC. Hence, we then used Gpx4 knockdown virus in the Hip to explore the role of Gpx4-related ferroptosis in the effects of EDA. Strikingly, EDA-induced antidepressant and anxiolytic effects were abolished. Unexpectedly, the object recognition memory was impaired after AAV–EGFP–Gpx4–miRNAi injection, indicating that Gpx4 may be associated with memory impairment.

Nevertheless, our study is not without limitations. First, the pathway from Sirt1 to Gpx4 is complex and we targeted only one portion of this pathway. Second, EDA alleviated neuroinflammation and neuronal loss in both the Hip and mPFC regions. Hence, we could not rule out the possibility that other mechanisms in the mPFC may underlie the functions of EDA. Third, although we demonstrated Gpx4 plays a key role in the effects of EDA, further research is required to investigate the underlying mechanisms of Gpx4-related ferroptosis in depression and anxiety.

## Conclusion

In summary, our study demonstrates for the first time that EDA could ameliorate depressive and anxiety-like behaviors, OS and neuroinflammation in a CSDS-induced mice model of depression. The underlying molecular mechanism may involve Gpx4-mediated ferroptosis via Sirt1/Nrf2/HO-1 pathway.

These results also highlight aberrant expression of Gpx4 as a potential mechanism in depression and suggest that Gpx4 mediated ferroptosis may be a promising new target for the treatment of MDD.

## Supplementary Information

**Additional file 1: Figure S1.** Ultrastructure of microglia in the CSDS model of Hip and mPFC with EDA treatment. **a** Electron micrographs showed mitochondrial damages in the hippocampal microglia (red arrows). Scale bars, 2 μm (upper panel) and 500 nm (lower panel). **b** Electron micrographs showed mitochondrial damages in the mPFC microglia (red arrows). Scale bars, 2 μm (upper panel) and 500 nm (lower panel).

**Additional file 2: Figure S2.** Pathway analysis of metabolomic alternations induced by CSDS and EDA treatment. IPA was conducted to identify the top canonical signaling pathways affected in CSDS mice with or without concomitant EDA treatment.

**Additional file 3: Figure S3.** Effect of EDA on Sirt1/Nrf2/HO-1/Gpx4 pathway in mRNA expression levels. **a**–**h** mRNA expression of Sirt1 (**a**), Nrf2 (**b**), HO-1 (**c**) and Gpx4 (**d**) in the hippocampal region. **e**–**h** mRNA expression of Sirt1 (**e**), Nrf2 (**f**), HO-1 (**g**) and Gpx4 (**h**) in the mPFC region. Data are presented as mean ± SEM (*n* = 8 per group). *p < 0.05, **p < 0.01   versus the CON + Vehicle group. ^#^*p* < 0.05, ^###^*p* < 0.001 versus the CSDS + Vehicle group.

## Data Availability

All data generated in this study are included in this manuscript.

## Abbreviations

OS: Oxidative stress
EDA: Edaravone
CSDS: Chronic social defeat stress
MDD: Major depression disorder
SI: Social interaction
SPT: Sucrose preference test
OFT: Open field test
EPM: Elevated plus maze
NOR: Novel object recognition
TST: Tail suspension test
FST: Forced swim test
TEM: Transmission electron microscopy
WB: Western blotting
qRT-PCR: Quantitative real-time polymerase chain reaction
AAV: Adeno-associated virus
Hip: Hippocampus
mPFC: Medial prefrontal cortex
RNS: Reactive nitrogen species
LTP: Long-term potentiation
Sirt1: Silent information regulator 2 homolog 1
Nrf2: Nuclear factor erythroid 2-related factor 2
HO-1: Heme oxygenase-1
Gpx4: Glutathione peroxidase 4
FDA: Food and Drug Administration
ALS: Amyotrophic lateral sclerosis
CON: Control
IZ: Interaction zone
MDA: Malondialdehyde
SOD: Superoxide dismutase
GSH: Glutathione
ANOVA: Analysis of variance
IPA: Ingenuity Pathway Analysis
AMP: Adenosine monophosphate
GMP: Guanosine monophosphate
GDP: Guanosine diphosphate
ADP: Adenosine diphosphate
KEGG: Kyoto Encyclopedia of Genes and Genomes
CUMS: Chronic unpredictable mild stress
BBB: Blood-brain barrier
GFAP: Glial fibrillary acidic protein
Iba-1: Ionized calcium-binding adaptor molecule 1
PBS: Phosphate-buffered solution
PC: Passive coping
AD: Alzheimer’s disease
BSA: Bovine serum albumin
CA3: Cornu ammonis 3
CX30: Connexin 30
CX43: Connexin 43
DG: Dentate gyrus
ELISA: Enzyme-linked immunosorbent assay
4-HNE: 4-hydroxynonenal
NADP: Nicotinamide adenine dinucleotide phosphate
8-OH-dG: 8-hydroxydeoxyguanosine
ROS: Reactive oxygen species
T-AOC: Total antioxidant capacity
TBA: Thiobarbituric acid
TBARS: Total antioxidant capacity
TREM2: Triggering receptor expressed on myeloid cells 2
