# Supplementary figures and images for: Edaravone ameliorates depressive and anxiety-like behaviors via Sirt1/Nrf2/HO-1/Gpx4 pathway
Source: J Neuroinflammation. 2022 Feb 7;19:41. doi: 10.1186/s12974-022-02400-6 (PMC8822843; doi:10.1186/s12974-022-02400-6)

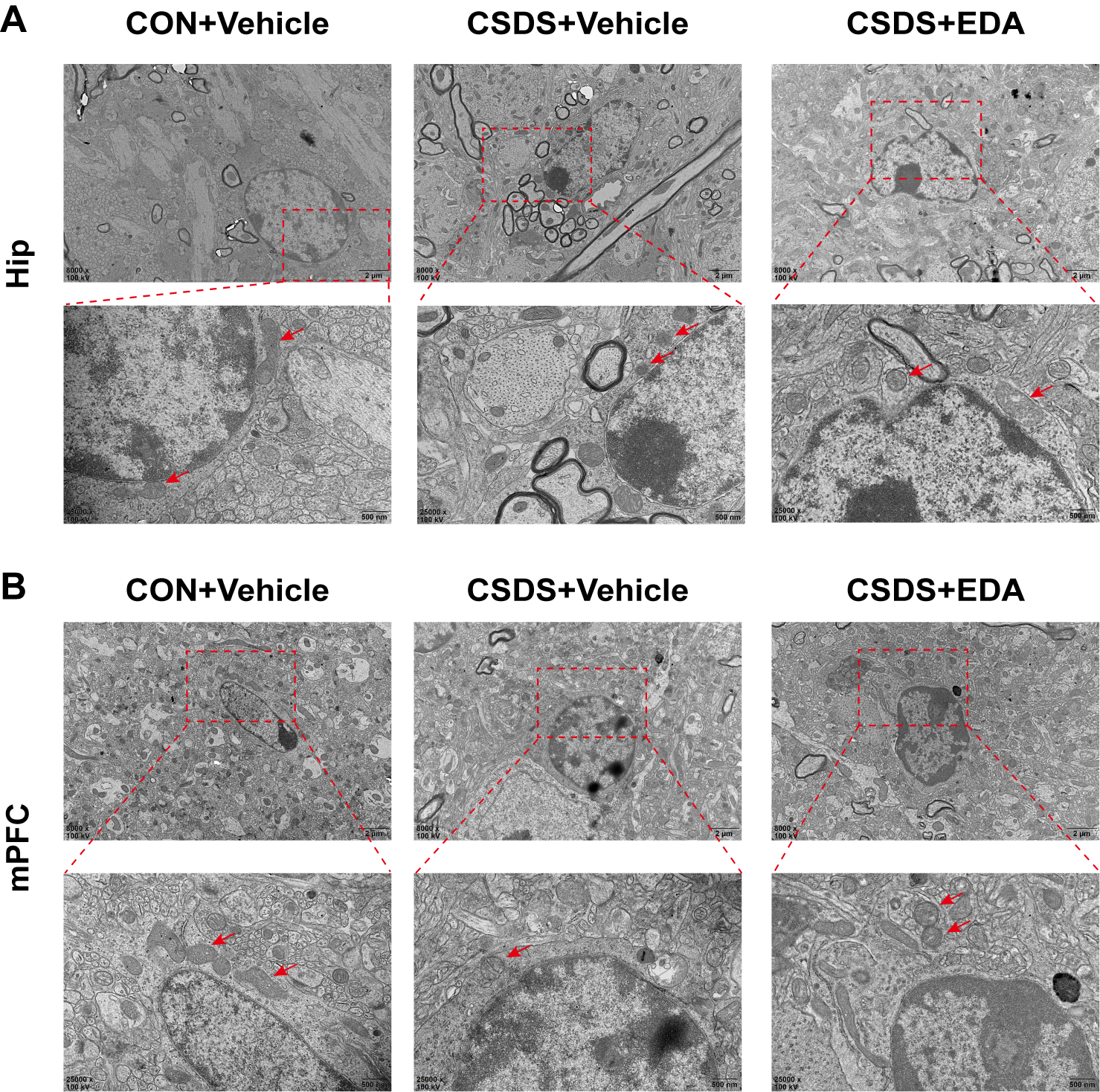

Supplement: Supplementary file 1 — Additional file 1: Figure S1. Ultrastructure of microglia in the CSDS model of Hip and mPFC with EDA treatment. a Electron micrographs showed mitochondrial damages in the hippocampal microglia (red arrows). Scale bars, 2 μm (upper panel) and 500 nm (lower panel). b Electron micrographs showed mitochondrial damages in the mPFC microglia (red arrows). Scale bars, 2 μm (upper panel) and 500 nm (lower panel). [file 12974_2022_2400_MOESM1_ESM.tif]

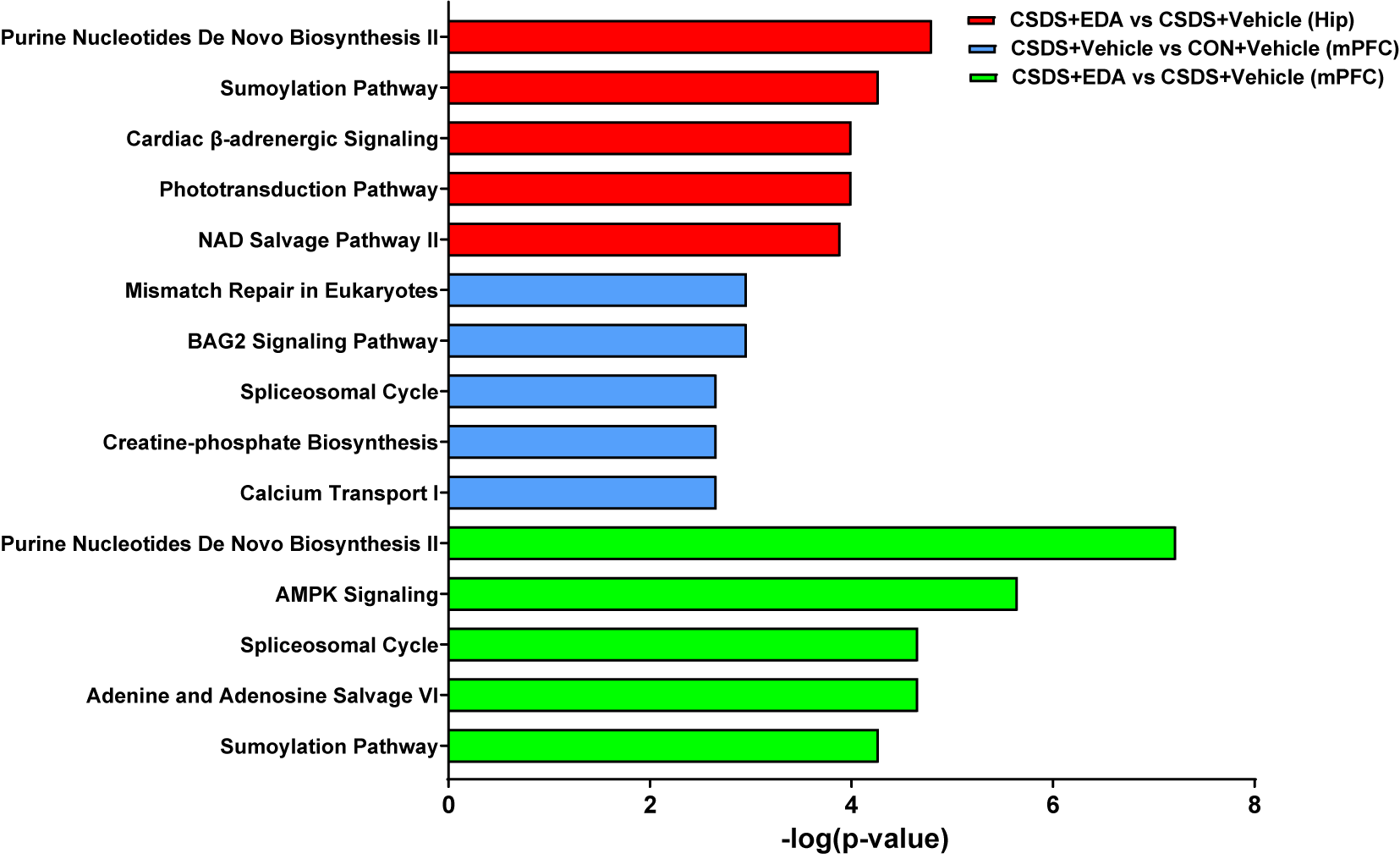

Supplement: Supplementary file 2 — Additional file 2: Figure S2. Pathway analysis of metabolomic alternations induced by CSDS and EDA treatment. IPA was conducted to identify the top canonical signaling pathways affected in CSDS mice with or without concomitant EDA treatment. [file 12974_2022_2400_MOESM2_ESM.tif]

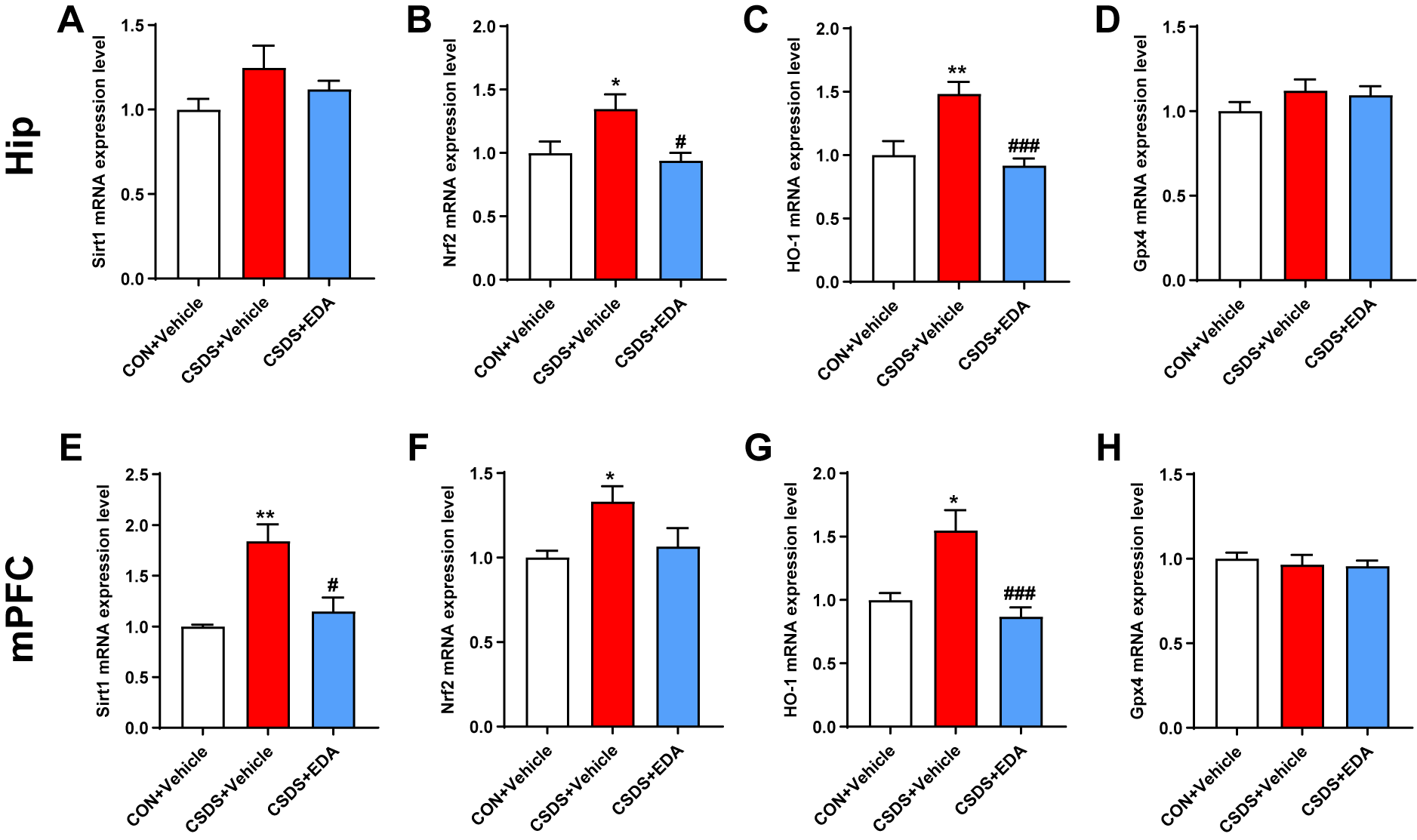

Supplement: Supplementary file 3 — Additional file 3: Figure S3. Effect of EDA on Sirt1/Nrf2/HO-1/Gpx4 pathway in mRNA expression levels. a–h mRNA expression of Sirt1 (a), Nrf2 (b), HO-1 (c) and Gpx4 (d) in the hippocampal region. e–h mRNA expression of Sirt1 (e), Nrf2 (f), HO-1 (g) and Gpx4 (h) in the mPFC region. Data are presented as mean ± SEM (n = 8 per group). *p < 0.05, **p < 0.01 versus the CON + Vehicle group. #p < 0.05, ###p < 0.001 versus the CSDS + Vehicle group. [file 12974_2022_2400_MOESM3_ESM.tif]
